# Supplementary material for: Breaking the burst: Unveiling mechanisms behind fragmented network bursts in patient-derived neurons
Source: Stem Cell Reports. 2024 Oct 3;19(11):1583–97. doi: 10.1016/j.stemcr.2024.09.001 (PMC11589196; doi:10.1016/j.stemcr.2024.09.001)
Supplement: Document S1. Figures S1–S4 and Table S1 [file mmc1.pdf]

Stem Cell Reports, Volume *19*

Supplemental Information

Breaking the burst: Unveiling mechanisms behind fragmented network bursts in patient-derived neurons

Nina Doorn, Eva J.H.F. Voogd, Marloes R. Levers, Michel J.A.M. van Putten, and Monica Frega

Supplemental Figures

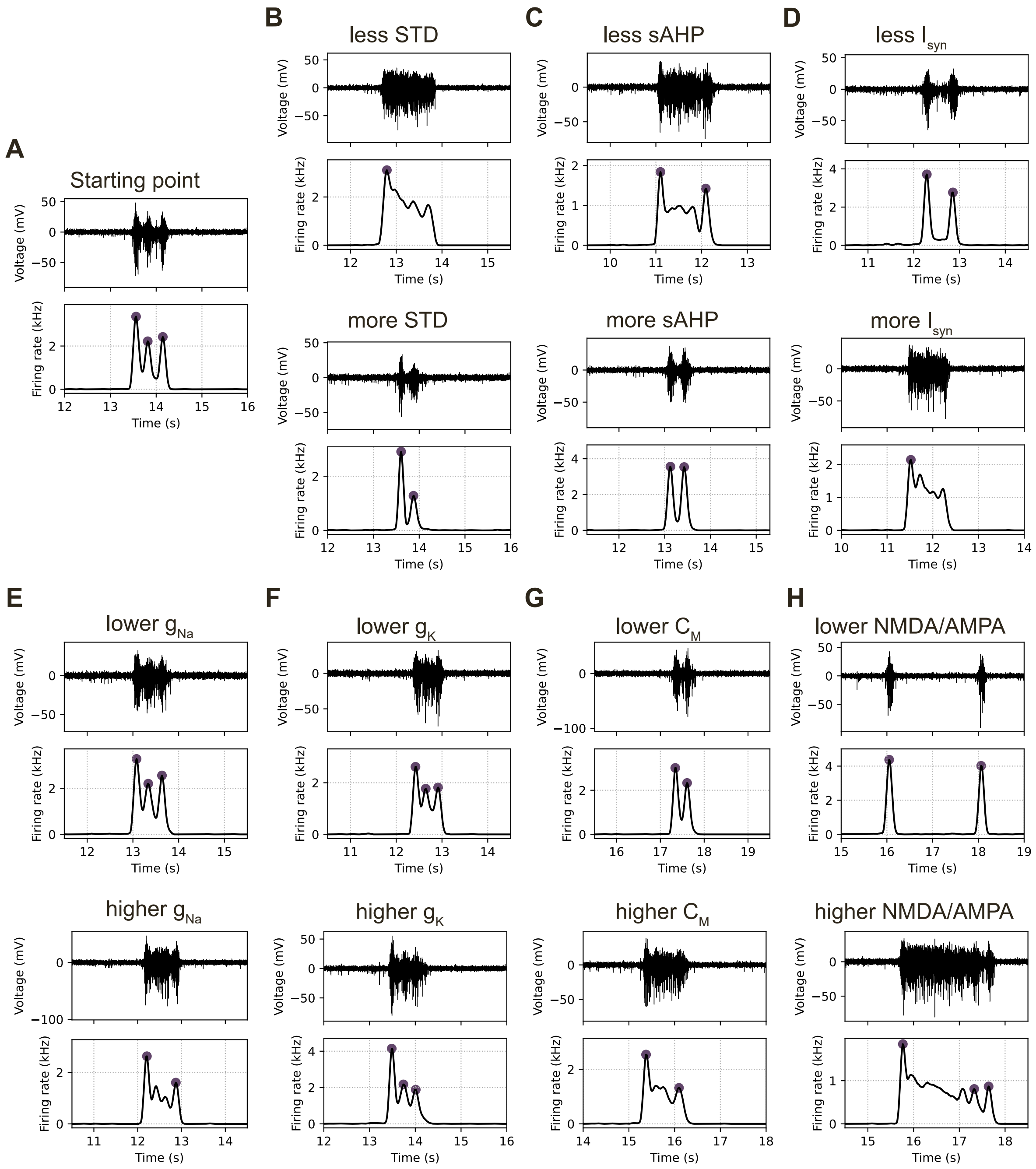

Figure S1: **The effect of model parameters on the number and shape of fragments in simulations.**
A) Starting point of parameter configuration resulting in simulations with network bursts (NBs) consisting of three fragments. **B)** The effect of less or more STD on the simulated NBs. Modeled as an increase or decrease in parameter U. **C)** The effect of less or more sAHP current on the simulated NBs. Modeled as an increase or decrease in the conductance of the sAHP channels. **D)** The effect of less or more synaptic current (I_{syn}) on the simulated NBs. Modeled as an increase or decrease in the conductance of all synaptic channels. **E)** The effect of a higher or lower maximum voltage-gated sodium channel conductance on the simulated NBs. **F)** The effect of a higher or lower maximum voltage-gated potassium channel conductance on the simulated NBs. **G)** The effect of a higher or lower neuronal membrane capacitance on the simulated NBs. **H)** The effect of a higher or lower NMDA/AMPA ratio on the simulated NBs. Modeled as a shift in the relative maximum conductances of the NMDA and AMPA channels (corresponding to the relative amount of channels or their synaptic strengths).

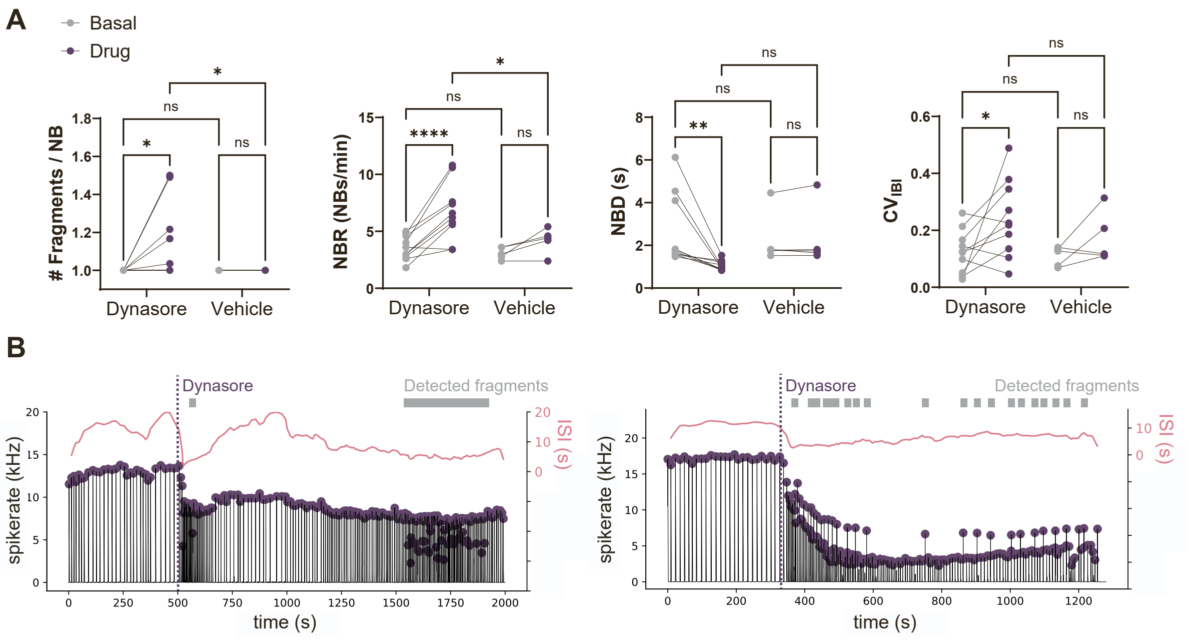

Figure S2: **Dynasore affects neuronal network activity differently from vehicle and the effect varies throughout measurement.**

A) The effect on the neuronal network activity when either Dynasore (10 μM) (n=10) or vehicle (0.1% DMSO) (n=5) is applied to the network. Figure shows the change in the average number of fragments per network burst (NB) (# Fragments / NB), the NB rate (NBR), the NB duration (NBD), and the coefficient of variation of the inter burst intervals (CV_{IBI}). ns $P>0.05$, * $P<0.05$, ** $P<0.01$, **** $P<0.0001$, 2way ANOVA with uncorrected Fisher's LSD for multiple comparisons was performed between groups. **B)** Two examples of recordings of healthy neuronal network activity in which Dynasore was added (dashed line). Shown is the network firing rate in black with detected peaks in purple. In both examples, a decrease in the network firing rate can be seen upon Dynasore application, together with a decrease in the inter-spike interval (ISI, pink line) and some fragment detections (grey bar). Then the ISI slowly increases again while the amount of fragment detections drops to zero. Towards the end of the recording, the amount of fragment detections again increases, illustrating the variable effect Dynasore has on the *in vitro* neuronal network over time.

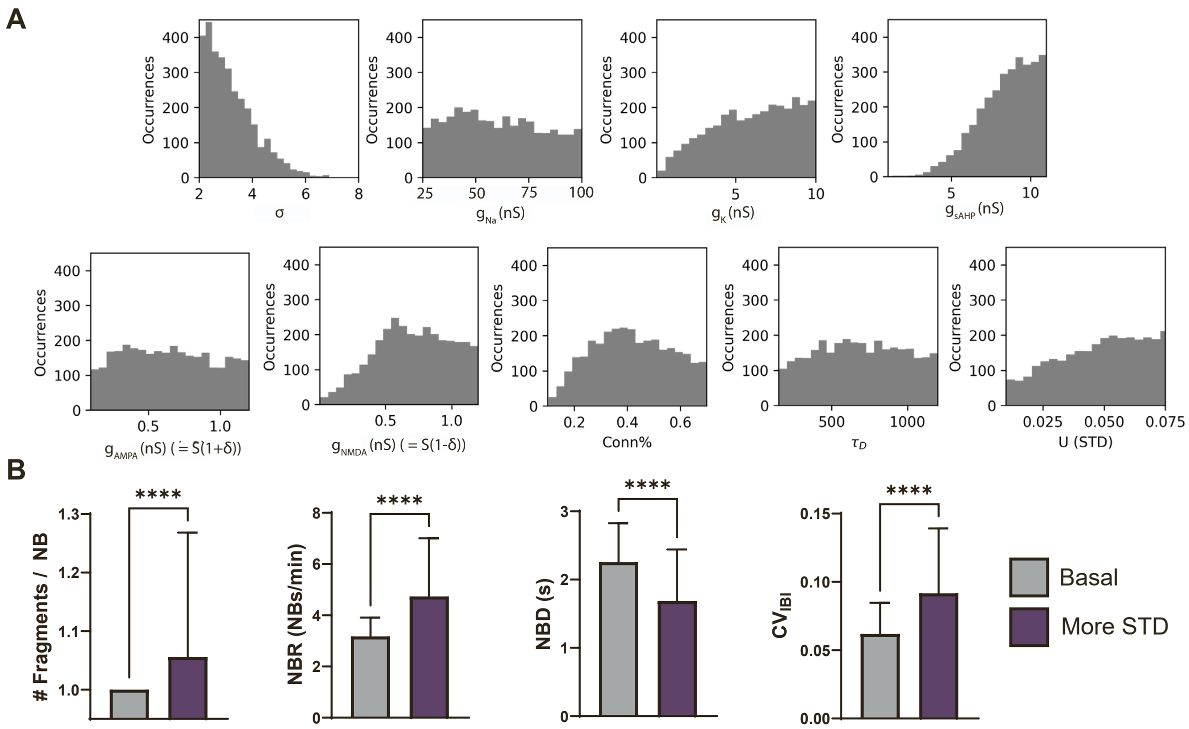

Figure S3: **The effect of increasing STD *in silico* with different starting parameter configurations.**

A) Histograms of the parameter values resulting in simulations consistent with experimental data. **B)** Grey shows data features of 3114 basal simulations with different model parameter configurations. Purple shows data features when the strength of STD (U) was increased by 50% in these simulations. Data shows mean $\pm$ sd. Wilcoxon matched-pairs signed rank test was performed between groups. **** P$<$0.0001.

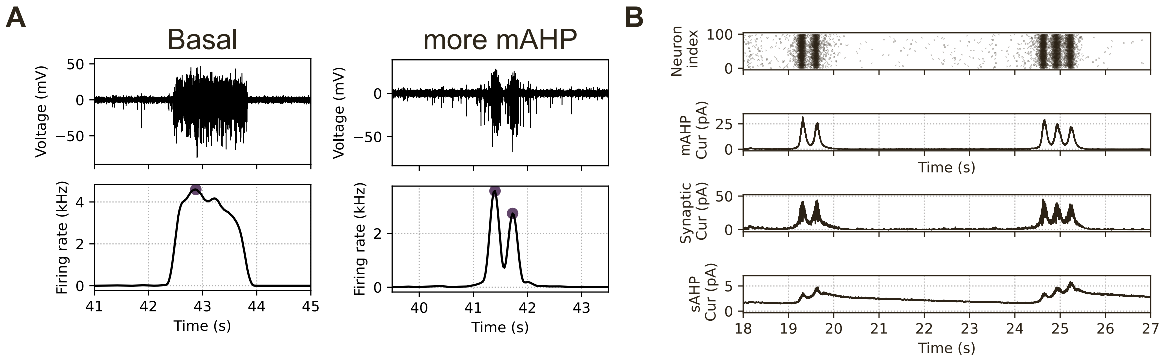

Figure S4: **Fragmented NBs can also arise from the interplay between mAHP and sAHP.** **A)** Top: Voltage recorded at a single virtual electrode. Bottom: Network firing rate with detected fragments (purple dots). Left: Basal situation with no STD and no mAHP. Right: Simulation of the same network but with increased mAHP. **B)** Mechanism causing fragmented NBs: Recurrent excitation (third panel) starts an NB causing an increase in firing (top panel). This increase in firing causes an increase in both the mAHP (second panel) and sAHP (fourth panel) currents. This lowers the firing rate, terminating the fragment. Then, mAHP recovers quickly, allowing left-over excitation to initiate the next fragment until sAHP (recovering slowly) is high enough to terminate the entire NB. mAHP is modeled identically to sAHP (conductance increases at every spike and decays exponentially), but with a time constant of 50 ms instead of 8000 ms and a conductance of 350 nS instead of 6 nS.

Supplemental Tables

Table S1: Values of the parameters of the realistic *in silico* model used for the results and examples shown in the Figures.

Parameter	Unit	Fig 2B	Fig 3C,D	Fig 4C,D	Fig 4G,H	Fig 4I,J
Connection prob		0.2	0.2	0.2	0.3	0.2
Membrane area	μm^{-2}	300	300	300	300	300
C	μF $\cdot$ cm^{-2}	2	2	2	1	2
E_{L}	mV	-39.2	-39.2	-39.2	-39.2	-39.2
E_{K}	mV	-80	-80	-80	-80	-80
E_{Na}	mV	70	70	70	70	70
g_{Na}	mS $\cdot$ cm^{-2}	80	80	80	50	80
g_{K}	mS $\cdot$ cm^{-2}	6.5	6.5	6.5	5	6.5
g_{L}	mS $\cdot$ cm^{-2}	0.3	0.3	0.3	0.3	0.3
Firing threshold	mV	-30.4	-30.4	-30.4	-30.4	-30.4
σ	mV	8	6	5.5	3.5	5.5
g_{sAHP}	nS	5	4	8	5	5
τ_{AHP}	ms	8000	8000	8000	8000	8000
α_{Ca}		0.00035	0.00035	0.00035	0.00035	0.00035
S		0.4	0.25	0.4	1.5	13
δ		0.6	0.6	0.6	0.5	0.6
E_{AMPA}	mV	0	0	0	0	0
E_{NMDA}	mV	0	0	0	0	0
τ_{AMPA}	ms	2	2	2	2	2
$\tau_{\text{NMDA,rise}}$	ms	2	2	2	2	2
$\tau_{\text{NMDA,decay}}$	Ms	100	100	100	100	100
α	kHz	0.5	0.5	0.5	0.5	0.5
τ_{D}	ms	200	250	200	800	1000
U		0.2	6e-3/35e-3	0.01	0.2	0.005
Apply STF	t/f	FALSE	FALSE	FALSE	FALSE	TRUE
τ_{F}	ms	-	-	-	-	1000
Apply Asyn	t/f	FALSE	FALSE	TRUE	FALSE	FALSE
τ_{ar}	ms	-	-	700	-	-
U_{ar}		-	-	0.5	-	-
U_{max}	1/ms	-	-	5e-4/45e-4	-	-
X_0		-	-	5	-	-
