# Supplementary material for: Breaking the burst: Unveiling mechanisms behind fragmented network bursts in patient-derived neurons
Source: Stem Cell Reports. 2024 Oct 3;19(11):1583–97. doi: 10.1016/j.stemcr.2024.09.001 (PMC11589196; doi:10.1016/j.stemcr.2024.09.001)
Supplement: Document S2. Article plus supplemental information [file mmc3.pdf]

Stem Cell Reports
Article
ISSCR
OPEN ACCESS

Breaking the burst: Unveiling mechanisms behind fragmented network bursts in patient-derived neurons

Nina Doorn,[1,3,*] Eva J.H.F. Voogd,[1] Marloes R. Levers,[1] Michel J.A.M. van Putten,[1,2] and Monica Frega[1]
[1]Department of Clinical Neurophysiology, University of Twente, Enschede 7522 NB, the Netherlands
[2]Department of Neurology and Clinical Neurophysiology, Medisch Spectrum Twente, Enschede 7512 KZ, the Netherlands
[3]Lead contact
*Correspondence: n.doorn-1@utwente.nl
https://doi.org/10.1016/j.stemcr.2024.09.001

SUMMARY

Fragmented network bursts (NBs) are observed as a phenotypic driver in many patient-derived neuronal networks on multi-electrode arrays (MEAs), but the pathophysiological mechanisms underlying this phenomenon are unknown. Here, we used our previously developed biophysically detailed *in silico* model to investigate these mechanisms. Fragmentation of NBs in our model simulations occurred only when the level of short-term synaptic depression (STD) was enhanced, suggesting that STD is a key player. Experimental validation with Dynasore, an STD enhancer, induced fragmented NBs in healthy neuronal networks *in vitro*. Additionally, we showed that strong asynchronous neurotransmitter release, NMDA currents, or short-term facilitation (STF) can support the emergence of multiple fragments in NBs by producing excitation that persists after high-frequency firing stops. Our results provide important insights into disease mechanisms and potential pharmaceutical targets for neurological disorders modeled using human induced pluripotent stem cell (hiPSC)-derived neurons.

INTRODUCTION

Human induced pluripotent stem cell (hiPSC)-derived neurons have emerged as an effective platform for drug screening and modeling of neurological disorders *in vitro*. When plated on multi-electrode arrays (MEAs), these neurons form functionally connected and spontaneously active networks (Mossink et al., 2021). This activity self-organizes into network bursts (NBs), which are drastic transient increases in spiking frequency occurring synchronously throughout the network. The properties of these NBs are often used for phenotypic characterization as they correlate with specific disease states (Van Hugte et al., 2023; Klein Gunnewiek et al., 2021; Klein Gunnewiek et al., 2020; Frega et al., 2019; Marchetto et al., 2017). Several of these genotype/phenotype correlations have been established by characterizing NBs, providing insight into the pathophysiological mechanisms underlying the neuronal network phenotype (Doorn et al., 2023; Klein Gunnewiek et al., 2021; Klein Gunnewiek et al., 2020; Frega et al., 2019; Marchetto et al., 2017; Linda et al., 2022; Wang et al., 2022).

While in healthy neuronal networks, NBs show a monotonous decrease in spiking frequency back to baseline activity, NBs in patient-derived neuronal networks may express prominent fluctuations in spiking frequency within this period, resulting in NBs consisting of multiple fragments (*fragmented* NBs). In research using neuronal networks derived from hiPSCs of patients with Dravet syndrome (DS), generalized epilepsy with febrile seizures plus (GEFS+), and febrile seizures with mutations in *SCN1A*, fragmented NBs were the main phenotypic driver (Van Hugte et al., 2023). Anti-epileptic drugs (AEDs) that were effective in those patients reduced the number of fragments per NB in their neuronal networks, while AEDs that exacerbated the clinical phenotype increased it. Similarly, in a model for Kabuki syndrome, a multisystem neurodevelopmental disorder (NDD), fragmented NBs were one of the main signs of altered network organization compared to healthy networks (Gabriele et al., 2021). Fragments in NBs were also visible in other NDD models such as Kleefstra syndrome (KS) (Frega et al., 2019) and Rett syndrome (RTT) (Pradeepan et al., 2024), and were suppressed with either NMDA receptor- or asynchronous neurotransmitter release blockers. However, how these processes contribute to the emergence of fragments remains unknown. Uncovering the complete pathways leading to the occurrence of fragmented NBs could help elucidate disease mechanisms at play in patient-derived neuronal networks and provide possible pharmaceutical targets.

Computational models can provide insight into the mechanisms underlying specific electrophysiological behavior. We previously developed a biophysically detailed computational model of hiPSC-derived neuronal networks on MEAs that can faithfully simulate the pattern of activity of healthy and patient-derived neuronal networks (Doorn et al., 2023). Here, we use this model to investigate the possible mechanisms underlying fragmented NBs.

Fragmented NBs in our model simulations occurred only when the level of short-term synaptic depression (STD) was substantial, suggesting this is a key player. We validated our hypothesis by increasing STD in healthy neuronal

Published by Elsevier Inc. on behalf of International Society for Stem Cell Research.
This is an open access article under the CC BY license (http://creativecommons.org/licenses/by/4.0/).

networks *in vitro* with Dynasore, which resulted in the emergence of fragmented NBs. Furthermore, we showed that enhanced STD in combination with persistent excitation supports the generation of multiple fragments in NBs. Our study provides crucial insights into disease mechanisms at play in neuronal network models of neurological disorders.

RESULTS

Fragmented NBs as a common phenotype in patient-derived neuronal networks

Fragmented NBs are observed in multiple patient-derived neuronal networks grown on MEAs. To illuminate the extent of this phenotype, we gathered MEA measurements of such networks. Specifically, we re-analyzed data from networks derived from patients with GEFS+ and DS (mutation in *SCN1A*) (Van Hugte et al., 2023), and KS (mutation in *EHMT1*) (Frega et al., 2019), as well as the corresponding healthy control networks. In all cases, patient-derived stem cells were differentiated into excitatory neurons through forced *Ngn2* overexpression (Figure 1A). The activity of all neuronal networks consisted of random spiking and NBs. Compared to controls, patient-derived neuronal networks showed altered NB characteristics, which were disease specific (i.e., more or less frequent, shorter or longer NBs, Figure 1B). Upon examination of recordings from a single electrode during NBs, it becomes apparent that these phenotypes have a shared characteristic, namely the presence of NBs consisting of fragments, resulting from prominent fluctuations in the spiking frequency within the NB (Figure 1C, top panels). The number and "shapes" of these fragments vary widely between these disorders. To quantify the number of fragments, we detected the local maxima in the smoothed network firing rate for each NB (see Methods for details), indicated with the colored dots in Figure 1C, bottom panels. We observed significantly increased numbers of fragments in patient-derived networks compared to control (Figure 1D). Thus, fragmented NBs were a common phenotype of patient-derived neuronal networks on MEAs.

Our biophysical models replicate fragmented NBs with increased STD

Next, we aimed to investigate the mechanisms underlying the appearance of fragmented NBs in patient-derived neuronal networks. For this, we employed our previously developed computational model of hiPSC-derived excitatory neuronal networks on MEA (Doorn et al., 2023), which has been shown to successfully identify the effect of specific cellular changes on the network activity.

Our biophysical model (Figure 2A) consists of heterogeneous Hodgkin-Huxley (HH) neurons, connected through synapses with AMPA and NMDA receptors (AMPARs and NMDARs), each with varying strengths and delays. The neurons contain voltage-gated potassium and sodium channels as well as slow-afterhyperpolarizing (sAHP) channels causing spike-frequency adaptation. Moreover, the synapses undergo STD: the amplitude of the excitatory postsynaptic current (EPSC) is depressed with every subsequent presynaptic spike and recovers quickly in the absence of spikes. The activity of the network is recorded by virtual electrodes, mimicking the experimental MEA recordings. The simulated activity of this model accurately recapitulated the NB characteristics of control neuronal networks at the single-electrode level (Figure 2B "Basal"; Figure 1C "Control").

Fragmented NBs can be simulated with this model by increasing the intensity of STD compared to simulations of healthy controls, resembling fragmented NBs observed *in vitro* (Figure 2B "FB ex1"; Figure 1C). The number and shapes of the fragments could subsequently be modified by additional changes in other model parameters, allowing *in silico* reproduction of the divergent *in vitro* phenotypes (Figure 2B "FB ex2" and "FB ex3"; Figure 1C). In particular, decreased sAHP currents, increased synaptic strengths or time constants, increased NMDA/AMPA ratio, increased membrane capacitance, or decreased STD resulted in more, but less defined fragments (Figure S1). Instead, changes in voltage-gated sodium and potassium channel conductances had very little effect on the fragments. Most importantly, sufficient STD (i.e., strong enough to significantly lower the firing activity) was crucial for the emergence of fragmented NB in all simulations.

Since our *in silico* model consists of many intricate and interacting mechanisms, we could not rule out whether mechanisms other than STD might be involved in the generation of fragmented NBs. To this end, we constructed a simpler computational model only containing the mechanisms we hypothesized to be necessary and sufficient for the emergence of NBs and fragments (i.e., sAHP, recurrent excitation, and STD). In this *minimal in silico* model (Figure 2C), neurons are represented by simple exponential integrate-and-fire (EIF) neurons including the sAHP current. Synapses contain generic synaptic receptors that immediately open in response to a presynaptic spike and undergo STD; further, all neurons and synapses are identical.

Also with this minimal model, we were able to obtain NBs with and without fragments, and we could identify the mechanisms leading to this phenomenon (Figure 1D). In the simulations, NBs without fragments (Figure 1E, "Basal") result from the interplay between recurrent excitation (I_{syn}) and sAHP (I_{AHP}), which suppress the activity of the neurons. Excitation starts the NB, and the rapid

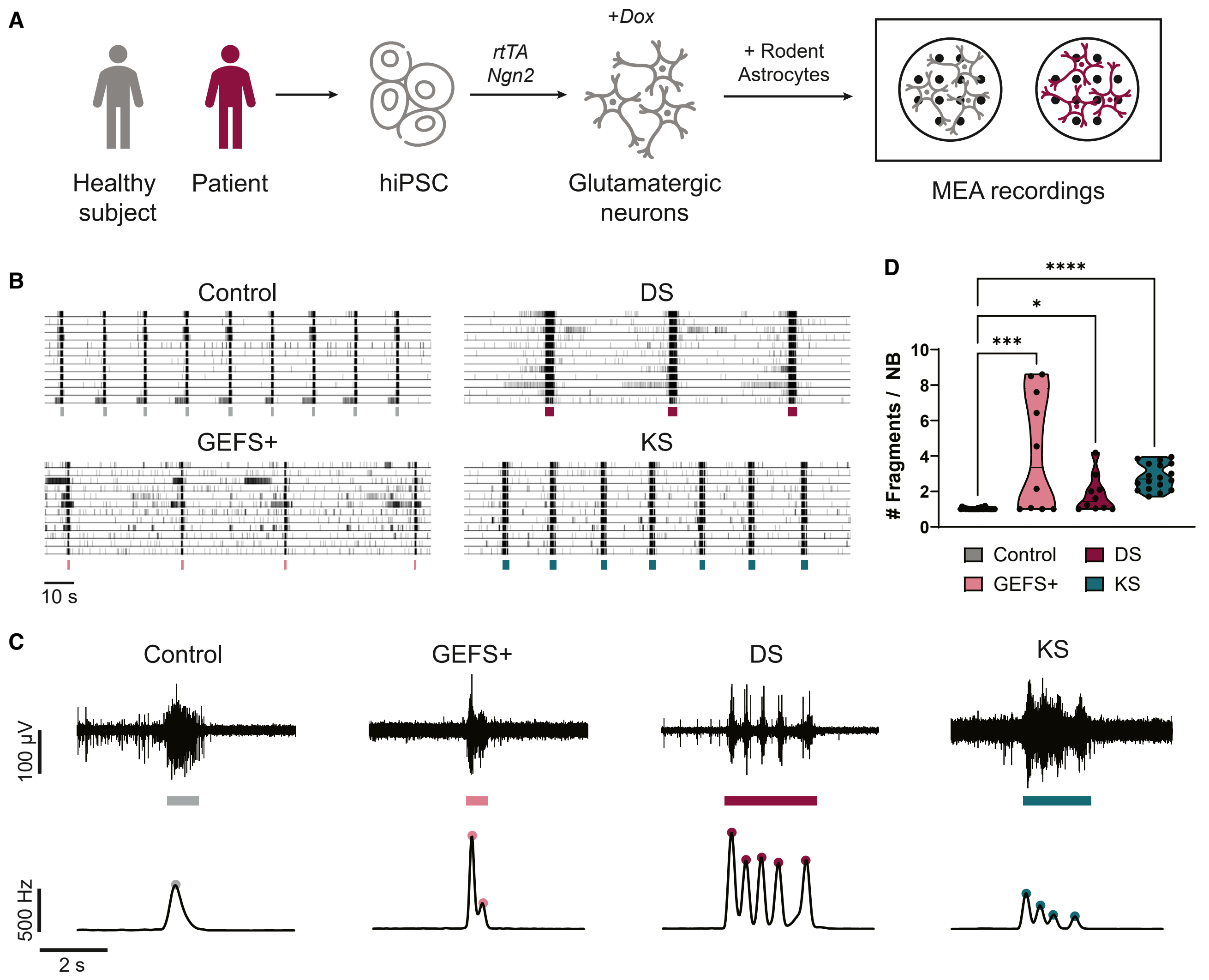

Figure 1. Fragmented network bursts in patient-derived neuronal networks on MEA

(A) Schematic overview of the protocol used by Van Hugte et al., (2023); Frega et al., 2019 to differentiate hiPSCs into neuronal networks on multi-electrode arrays (MEAs). hiPSCs were obtained by reprogramming somatic cells of healthy subjects and patients. Excitatory neurons were generated through doxycycline (Dox)-inducible overexpression of Neurogenin2 (*Ngn2*). At day *in vitro* (DIV) 2, E18 rat astrocytes were added in a 1:1 ratio. Activity was recorded at DIV 35 with MEA.

(B) Representative raster plots of spontaneous activity of healthy control neuronal networks (Control) and networks derived from patients with GEFS+, Dravet Syndrome (DS), and Kleefstra Syndrome (KS) recorded by Van Hugte et al., (2023); Frega et al., 2019. Detected network bursts (NBs) are indicated by the colored bars below.

(C) Top: representative example voltage traces recorded at one electrode during an NB detection (bar below). Bottom: representative network firing rate traces during the same NB and detected fragments (colored dots).

(D) Quantification of the average number of fragments per NB for Control ($n = 26$), GEFS+ ($n = 10$), DS ($n = 11$), and KS ($n = 18$) networks. *$p < 0.05$, ***$p < 0.001$, ****$p < 0.0001$, Kruskal-Wallis test with Dunn's multiple comparisons test was performed between groups.

firing causes an increase in the sAHP current. As soon as sAHP is stronger than excitation, the NB stops. Then, sAHP recovers slowly until it is low enough for excitation to initiate the next NB. In simulations with fragmented NBs (Figure 1E, "FB"), STD is strong enough to depress the synapses when rapid firing starts, thereby lowering the firing rate and halting the increase in sAHP. STD then quickly recovers, allowing excitation to become stronger again, and the next fragment starts. This continues until sAHP overcomes excitation and terminates the entire NB. The number of fragments is determined by how fast sAHP overcomes excitation, with more fragments occurring when excitation is stronger than adaptation for longer periods. Our realistic and minimal

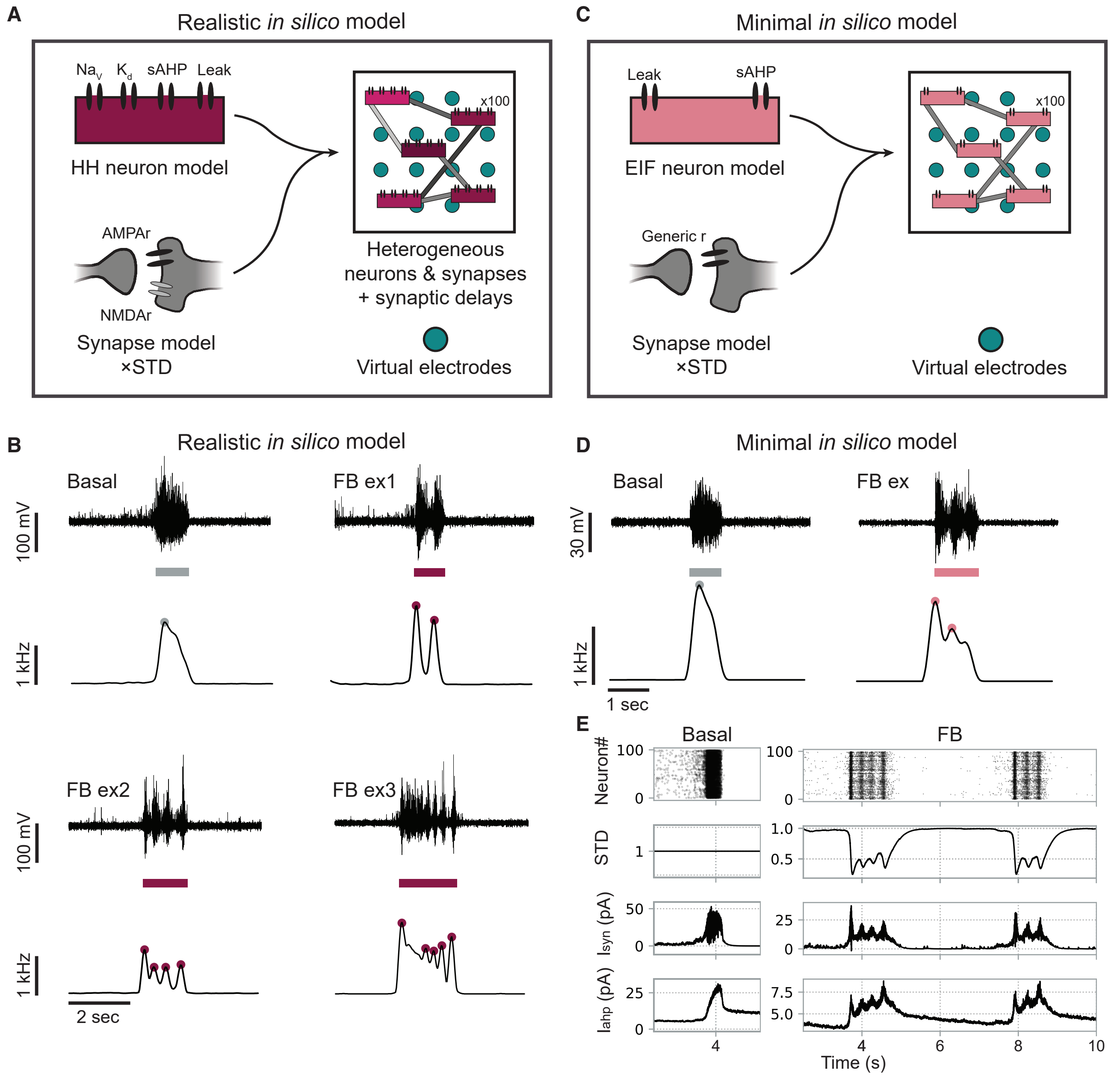

Figure 2. Fragmented network bursts can be replicated in *in silico* models with sufficiently strong short-term synaptic depression

(A) Schematic representation of the realistic *in silico* model consisting of 100 Hodgkin-Huxley (HH) type neurons with voltage-gated potassium and sodium channels, slow-afterhyperpolarizing (sAHP) and leaky currents, connected with AMPA receptor (AMPAr) and NMDA receptor (NMDAr) synapses, including short-term synaptic depression (STD). All neurons are heterogeneously excitable, with varying synaptic strengths and delays, forming a two-dimensional network. Virtual electrodes are employed to simulate MEA electrodes.

(B) Simulations with the realistic computational model show typical network bursts (NBs) ("Basal") or, as STD is increased, fragmented NBs ("FB example ex1"). Subsequently, different kinds of fragmented NBs can be created by altering the sAHP and synapse properties (FB examples ex1–ex3). Top: shows voltages recorded at one virtual electrode during a detected NB (colored bar below); bottom: shows the corresponding network firing rate with detected fragments (colored dots).

(C) Minimal *in silico* model with 100 homogeneous exponential integrate-and-fire (EIF) neurons including the sAHP current, homogeneously connected through synapses with generic post-synaptic receptors and STD.

(D) Simulations with the minimal model can also show typical and fragmented NBs.

(legend continued on next page)

computational models thus suggest that sufficient STD induces fragmented NBs.

Enhancing STD *in vitro* induces fragmented NBs

Next, we aimed to test the hypothesis predicted by our *in silico* model that increased STD causes fragmented NBs by performing validation *in vitro*. To this end, we applied Dynasore (i.e., dynamin inhibitor that enhances STD (Hua et al., 2013)) to healthy neuronal networks that showed regular NBs. Upon applying Dynasore (10 μM), fragmented NBs emerged in 50% of the neuronal networks, with two fragments per NB (Figures 3A, 3B, and 3E).

To investigate why Dynasore only induced NBs with a maximum of two fragments, we modeled the effect of Dynasore in our realistic *in silico* model by increasing the amount of STD while leaving all other parameters unchanged. Highly similar to the *in vitro* observations, this induced the appearance of either two fragments or of a single fragment with very short NB duration (NBD) (Figures 3C–3E). We found that when a single fragment occurred, the strength of STD was larger compared to the strength of excitation—which differed per network due to random connectivity—such that sAHP could overcome excitation after the first fragment.

To better compare the *in vitro* effect of Dynasore to the *in silico* increase in STD, we defined additional features of network activity that were affected by Dynasore (Figure 3E). Specifically, the NBD significantly decreased in all wells, and the NB rate (NBR) and coefficient of variation of the inter-burst intervals (CV_{IBI}) significantly increased. These changes were not observed when applying a DMSO vehicle to the neuronal networks (Figure S2A). Identical to these *in vitro* observations, enhancing STD *in silico* caused an increase in the NBR and a decrease in the NBD. Only the CV_{IBI} did not significantly change *in silico*.

Since many other *in silico* model parameter configurations can also simulate *in vitro* neuronal network behavior, we investigated the influence of increasing STD in a wide range of these configurations (Figure S3A). Out of 120,000 tested parameter configurations, we identified 3,114 configurations resulting in simulations resembling basal *in vitro* activity. When increasing STD in these simulations, fragments appeared in 20% of the cases. About 1% showed more than two fragments. Consistent with previous *in silico* results, we found an increase in NBR and CV_{IBI} and a decrease in NBD in the vast majority of the simulated neuronal networks (Figure S3B).

To summarize, we show that fragmented NBs can be induced in healthy neuronal networks by enhancing the amount of STD with Dynasore and that the effect of Dynasore was highly similar to enhancing STD *in silico* in a large range of parameter configurations.

Persistent excitation in addition to enhanced STD induces multiple fragments

By only enhancing STD *in vitro*, we could obtain NBs with a maximum of two fragments and a short duration, while patient-derived neuronal networks may show NBs with many more fragments exceeding the NBD of healthy networks (Figure 1C). With our *in silico* models, multiple fragments could be simulated with enhanced STD and excitation stronger than adaptation for a prolonged time (Figure S1). Recently, Pradeepan et al. showed that these multiple fragments occurring in the NBs of neuronal networks derived from patients with RTT *in vitro* (Figures 4A and 4B) disappeared when blocking asynchronous neurotransmitter release (Pradeepan et al., 2024). However, it is unclear how asynchronous neurotransmitter release results in NBs with multiple fragments and whether it is associated with the mechanisms revealed by our *in silico* model.

To investigate these mechanisms, we implemented asynchronous transmitter release in our realistic *in silico* model using a phenomenological description (Wang et al., 2015). This asynchronous release model describes an increased probability of stochastic neurotransmitter release upon high-frequency pre-synaptic firing, utilizing the same vesicle pool as in "regular" synchronous release. With this model, NBs observed in control neuronal networks could be replicated with low amounts of asynchronous release, while the NBs with multiple fragments observed in diseased networks could be reproduced when the amount of asynchronous release was increased while leaving other parameters unaltered (Figures 4C and 4D). We could then identify the mechanisms leading to the occurrence of multiple fragments in NBs (Figures 4E and 4F). In the model, since asynchronous and synchronous release utilize the same vesicle pool, an increase in asynchronous release results in a more depressed synchronous release, and thus increased STD. Hence, fragmentation is still caused by increased STD. However, we noticed that asynchronous release persists long after the pre-synaptic neuron stops firing, allowing the next fragments to be induced after sAHP suppresses the firing. By observing the dynamics of sAHP and excitation, we saw that after every fragment the amount of sAHP is higher and it takes longer before

(E) Bursting mechanism resulting in basal or fragmented NBs. Recurrent excitation (I_{syn}, third) starts a burst causing rapid firing (top). This causes the sAHP current (I_{ahp}, fourth) to increase, hyperpolarizing the neurons and eventually terminating the NB. In fragmented NBs, the rapid firing causes STD (second) to kick in and lower the firing. STD then recovers quickly, allowing the remaining excitation to initiate the next fragment until the sAHP current is high enough to terminate the entire NB.

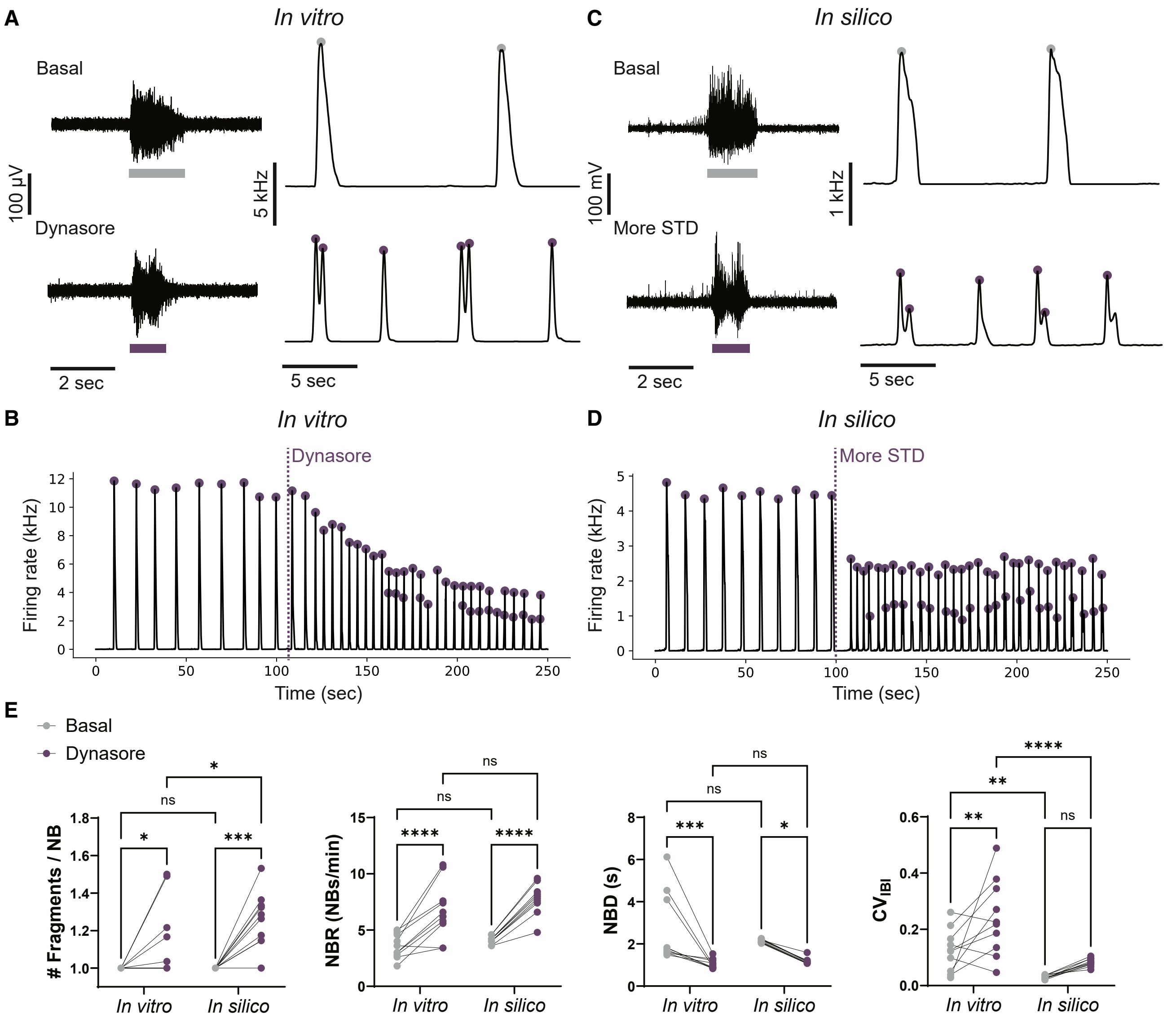

Figure 3. Dynasore induces fragmented network bursts in healthy neuronal networks similar to simulations with increased short-term depression

(A) Left: example voltage traces recorded at one electrode during a network burst (NB) detection (colored bar below) in a healthy neuronal network *in vitro* before (top) and after (bottom) application of Dynasore (10 μM). Right: example network firing rate traces before (top) and after (bottom) application of Dynasore (detected fragmented are indicated by colored dots).

(B) Representative network firing rate trace when Dynasore is applied to healthy neuronal networks *in vitro* at t = 105 s. Purple dots are detected fragments, when two dots appear during one increase in firing rate, two fragments are detected in one NB.

(C) Left: example voltage traces recorded *in silico* by one virtual electrode during a simulation with basal values for short-term synaptic depression (STD) (top) and increased values for STD (bottom). Right: example network firing rate traces of simulations with basal values for STD (top) and increased values for STD (bottom).

(D) Representative network firing rate trace when the amount of STD is increased *in silico* at t = 100 s.

(E) Quantification of the change when applying Dynasore *in vitro* ($n = 10$) and increasing STD *in silico* ($n = 10$) of the number of fragments per NB (# Fragments/NB), the NB duration (NBD), the NB rate (NBR), and the coefficient of variation of the inter burst intervals (CV_{IBI}). ns $p > 0.05$, *$p < 0.05$, **$p < 0.01$, ***$p < 0.001$, ****$p < 0.0001$, two-way ANOVA with uncorrected Fisher's LSD for multiple comparisons was performed between groups.

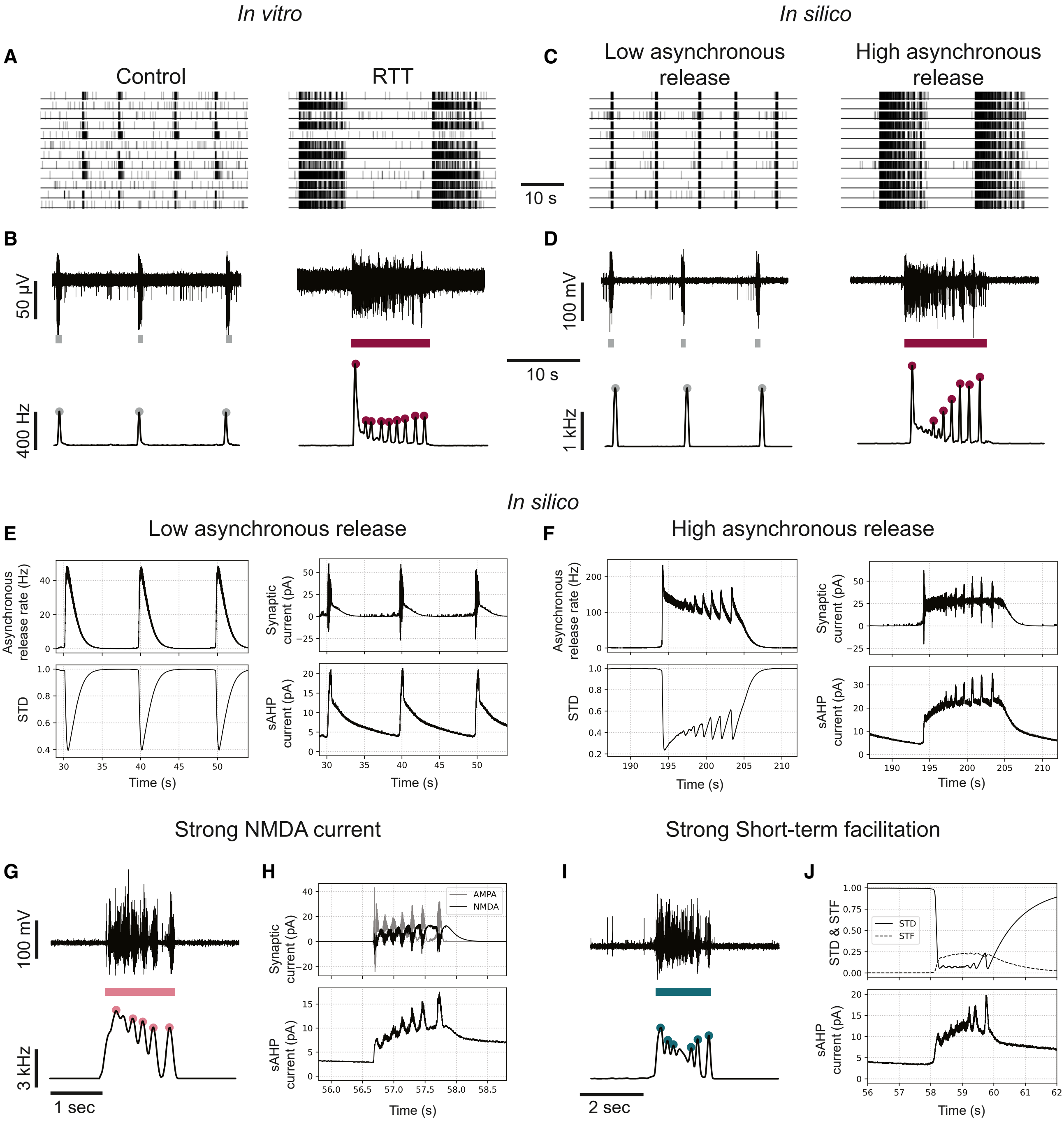

Figure 4. Multiple fragments can be generated *in silico* by enhanced STD with asynchronous release, NMDA currents, or short-term facilitation

(A) Representative raster plots of spontaneous neuronal network activity of healthy neuronal networks (left) and neuronal networks derived from a patient with Rett syndrome (RTT), measured by Pradeepan et al., (2024).

(B) Example voltage traces recorded at one electrode in the healthy network (top left) or RTT network (top right) during network burst (NB) detections (colored bar below), and example network firing rate traces during the same period (bottom) showing fragments (colored dots).

(C) Representative raster plots of simulated activity with low amounts of asynchronous release (left) and high amounts of asynchronous release (right).

(legend continued on next page)

excitation can overcome sAHP to initiate the next fragment, sometimes causing the slowing of the fragments. But, because STD can recover more in this longer period, the synapses will also be stronger with every fragment initiation, causing every subsequent fragment to be more synchronous compared to the last and thus have a larger firing rate amplitude.

Motivated by our hypothesis that multiple fragments are supported by excitation that persists after the pre-synaptic neuron stops firing, we investigated whether other mechanisms of persistent excitation could also support multiple fragment generation. First, since NMDArs open in response to post-synaptic depolarization and typically close slowly, they could cause left-over excitatory currents long after pre-synaptic firing activity stops (Hedegaard et al., 2012). We therefore modeled a strong NMDA current in our *in silico* model together with sufficient STD but without asynchronous release. This model indeed also showed NBs with multiple fragments (Figure 4G), where NMDA currents persisted after fragment termination, allowing the next fragment to be initiated (Figure 4H). Short-term synaptic facilitation (STF) can also cause neurotransmitter release after high-frequency firing has ended (Jackman and Regehr 2017). To explore this scenario, we constructed a model including STF and sufficient STD but without asynchronous release. In this model, we also observed multiple fragments (Figures 4I and 4J). Thus, persistent excitation after firing activity ceases supports the generation of multiple fragments. Note that in all cases, STD remains the mechanism allowing the emergence of fragments.

DISCUSSION

Fragmented NBs are observed in excitatory neuronal networks derived from patients with several neurological disorders (Van Hugte et al., 2023; Frega et al., 2019; Gabriele et al., 2021; Pradeepan et al., 2024). Different names are given to the phenomenon, such as mini-bursts, reverberating bursts, high-frequency bursts, and superbursts, but in essence, they are all characterized by a fluctuating spiking frequency within the NBs. In some experimental work, fragmented NBs were suppressed by either NMDAr blockers (Frega et al., 2019) or inhibition of asynchronous release (Pradeepan et al., 2024). However, none of these studies explain how those processes lead to the formation of fragmented NBs, limiting our understanding of disease mechanisms.

In this work, we aimed to identify candidate biological mechanisms involved in the generation of fragmented NBs, using a biophysical *in silico* model previously developed and validated by us (Doorn et al., 2023), complemented by *in vitro* validations of model predictions.

As confirmed by modeling studies (Masquelier and Deco 2013; Dao Duc et al., 2015; Fardet et al., 2018), we showed that elementary bursting in excitatory neuronal networks results from the interplay between excitation and adaptation, modulated by sAHP. Simulations with our biophysical *in silico* model—as well as with our reduced model containing only recurrent excitation, adaptation, and STD—predict that sufficient STD can lead to the emergence of fragmented NBs. This is similar to previous computational modeling work of excitatory primary cultures on MEA, where it was suggested that adaptation, STD, and STF are necessary for the emergence of "repeated network spikes" (Masquelier and Deco 2013). However, we show that only STD and adaptation, even without STF, are enough to get NBs with fragments, dissecting the mechanisms even further. Also, mechanisms proposed in previous work were not verified *in vitro* (Masquelier and Deco 2013). Here, we used Dynasore, a drug that enhances STD, to induce fragments *in vitro* and test our hypothesis.

Dynasore inhibits dynamin, a GTPase protein that is essential for endocytosis, and has been shown to inhibit

(D) Example voltage traces recorded at one virtual electrode in a simulation with low amounts of asynchronous release (top left) or high amounts of asynchronous release (top right) during NB detections (colored bar below), and example network firing rate traces during the same period (bottom) showing fragments (colored dots).
(E) NB mechanism in simulations with low amounts of asynchronous release: the burst ends because there is not enough asynchronous release to initiate the next fragment when sAHP terminates the NB. (F) NB mechanism in simulations with high amounts of asynchronous release: the firing rate decreases because asynchronous release depletes the amount of available neurotransmitters, but because sAHP is not high enough to terminate the NB and there is enough remaining asynchronous release, the next fragment is initiated, repeating itself until sAHP reaches a threshold.
(G) Top: representative voltage trace recorded at one virtual electrode during a simulation without asynchronous release but with strong NMDA currents and bottom: representative network firing rate trace during the same NB showing multiple fragments.
(H) Mechanism of multiple fragments: a burst fragment is terminated by short-term synaptic depression (STD), but because NMDA channels close slowly and only open when the postsynaptic neuron is depolarized, the NMDA current revives the burst causing the next fragment, repeating itself until the sAHP reaches a threshold.
(I) Top: representative voltage trace recorded at one virtual electrode during a simulation without asynchronous release but with short-term synaptic facilitation (STF) and bottom: representative network firing rate trace during the same NB showing persistent fragments.
(J) Mechanism of multiple fragments: a burst fragment is terminated by STD, but the burst is revived by STF.

synaptic vesicle recycling in hippocampal neurons in a dose-dependent manner (Newton et al., 2006). As a result, Dynasore enhances the amount of STD in these neurons (Hua et al., 2013). However, Dynasore might also have other effects on different types of neurons. In brainstem slices, Dynasore completely blocked evoked synaptic responses but increased the spontaneous EPSC (sEPSC) frequency (Hofmann and Andresen 2017). In the frog neuromuscular junction, Dynasore not only inhibited endocytosis but also led to an increased probability of neurotransmitter release and increased resting intra-terminal calcium (Douthitt et al., 2011). Nevertheless, adding Dynasore in our *in vitro* cultures had an effect that was highly similar to enhancing STD *in silico*, with two fragments emerging in some but not all networks, and with identical changes of the NBR and NBD. This suggests that in our hiPSC-derived excitatory cultures, Dynasore works by enhancing the amount of STD. Nevertheless, the effects *in vitro* and *in silico* showed some differences. While the CV_{IBI} significantly increased *in vitro*, the increase *in silico* was not significant, and the values were generally lower. Moreover, the magnitude of the increase in the average number of fragments was higher *in silico* than *in vitro*. Both of these discrepancies might be explained by the fact that the effect of Dynasore changed quite rapidly *in vitro*, while the effect of enhancing STD *in silico* was instant and constant (Figure S2B). The effect of Dynasore slowly increased after administration, and then decreased again, causing temporal variations in the number of fragments. At some time points, the effect of Dynasore was so strong that NBDs were shorter than one fragment, and synapses were presumably so depressed that more fragments could not be initiated. Also, inter-burst intervals varied with the magnitude of the effect of Dynasore, while this remained constant *in silico*, explaining the higher CV_{IBI} *in vitro*. Importantly, the simulations shown in Figure 3 are all generated with the same configuration of model parameters, while other configurations can also simulate basal *in vitro* neuronal network behavior. By performing simulations in a larger range of parameter configurations, we found that the increase in STD had a similar global effect: an increase in the number of fragments, NBR, and CV_{IBI} and a decrease in NBD. However, NBs consisting of more than two fragments appeared. This occurred in simulation with low initial adaptation, allowing multiple fragments to occur. In addition, we observed an increase of NBD in 5.5% of the simulations. This occurred when the initial adaptation and strength of STD were notably high, causing a non-linear interaction upon increasing the strength of STD.

In the neuronal networks in which Dynasore caused fragmented NBs, at most two fragments were seen. This was different from some phenotypes observed *in vitro*, where sometimes up to eight fragments were observed (Figure 1C). While the increase in STD allows the emergence of fragments due to a fast-recovering depression of synaptic transmission, it also allows adaptation to overcome the suppressed excitation faster, terminating the NB sooner. Thus, when only enhancing STD without further altering the excitation-adaptation balance, the NB will always become significantly shorter, and no additional fragments can be generated.

In recent work by Pradeepan et al., (2024), multiple fragments occurred in excitatory neuronal networks derived from patients with RTT. These multiple fragments could be blocked using EGTA-AM, suggesting they are caused by asynchronous neurotransmitter release. With our *in silico* models, we showed that the number of fragments in NBs can be increased if excitation dominates over adaptation for a prolonged time. However, how this relates to asynchronous neurotransmitter release is unknown. By implementing asynchronous release in our model, we were able to obtain NBs with multiple fragments in simulations. We showed that asynchronous neurotransmitter release induced NBs with multiple fragments through the same uncovered mechanism since it causes both enhanced STD and prolonged excitation. We were also able to simulate similar NBs using enhanced STD in combination with other mechanisms that prolong excitation, particularly strong NMDA currents, and STF.

NBs with multiple fragments are also observed as a relatively rare phenomenon in healthy neuronal networks with excitatory and inhibitory neurons dissociated from rodents (Wagenaar et al., 2006; Huang et al., 2017). These fragmented NBs are often referred to as reverberations and they usually show a defined shape. The first fragment has a high firing rate and high synchronicity and is followed by a drastic drop in both. Then, for every subsequent fragment, the firing rate and synchronicity increase, as well as the time between fragments (Huang et al., 2017). We also observed this slowing and growing shape in our simulations, where it was caused by increasing adaptation and fast-recovering STD.

Through patch-clamp technology, it has been observed that a single neuron in a network can also exhibit reverberating activity when stimulated with a brief pulse (Lau and Bi, 2005; Volman et al., 2007; Compte et al., 2000). In particular, Lau and Bi (Lau and Bi 2005) showed that evoked reverberations in excitatory-inhibitory cultures could be abolished by EGTA-AM, similar to the model of RTT, and that applying Strontium, which elevates asynchronous release, exacerbated reverberations. Moreover, Volman et al., (2007) showed, employing a computational excitatory network model, that evoked fragments are maintained by enhanced asynchronous transmitter release. Additionally, they showed that a fast-timescale depression is responsible for oscillations during NBs, and

a slow-timescale depression is responsible for the termination of the NBs. In our model, sAHP serves as a slow-timescale depression that also terminates the NBs. STD is a fast-timescale depression in our model and indeed causes oscillations within the NB, which we call fragments. This substantiates our hypothesis that STD, or another fast-timescale depression, is still necessary to get fragments in NBs. Volman et al. did not include NMDArs in their computational model and argued that, therefore, it was impossible to rule out that NMDA might play a similar role as asynchronous release. Lau and Bi found that complete blockage of NMDArs reduced the occurrence and duration of reverberations (Lau and Bi 2005). Using computational modeling, Wang (Wang 1999) and Compte et al. (Compte et al., 2000) found that to obtain reverberations in excitatory-inhibitory networks, recurrent excitatory synapses must be dominated by a slow component. With their simulations, they show that network dynamics are essentially asynchronous when NMDArs dominate over AMPArs. Conversely, when AMPArs dominate, the network displays coherent single NBs. Thus, NMDArs are often thought to be important in reverberatory/fragmented activity. Alternatively, Dao Duc et al. (Dao Duc et al., 2015) used hippocampal cultures and slices without inhibition, as well as computational modeling, to show that the interplay of STD and STF drives NBs with multiple fragments. It is important to note that while our work has been performed on neuronal networks composed of only excitatory neurons, some of the reported studies focus on neuronal networks that include inhibitory neurons, which can also modulate the phenotype (Lau and Bi 2005). Nevertheless, inhibition is not required to produce reverberations in these networks (Lau and Bi 2005), and interestingly, the identified players in those reverberations (STD, asynchronous release, NMDArs, and STF) were similar to the ones found in our model.

With our biophysical computational model that includes asynchronous release, NMDArs, and STF, we showed that all of those mechanisms allowed the generation of multiple fragments in NBs. However, when using realistic time constants for NMDAr dynamics and STF, NBs and fragments were generally shorter than in simulations with asynchronous release. Yet, the time constants for asynchronous release used here are not based on literature, as it is unknown for these types of cultures. Moreover, we assumed asynchronous and synchronous synaptic release both use the same neurotransmitter vesicle pool, even though these pools are also suggested to be different in some types of neurons (Kaeser and Regehr 2014). We found that if asynchronous release indeed uses the same vesicle pool, enhanced asynchronous release automatically results in more depressed synchronous release, and thus the emergence of fragments by enhanced STD. In this way, the difference between healthy cultures and RTT cultures from the study by Pradeepan et al., (2024) could be modeled solely by increasing the amount of asynchronous release. If both types of release use a different vesicle pool, an additional increase in STD would be needed to simulate the phenotype, as was necessary for simulations of NBs with multiple fragments with NMDArs and STF.

With our results, we can summarize and generalize previous research by showing that NBs with multiple fragments can be caused by (1) sufficient STD (or another fast-timescale depression that is able to temporarily lower the firing rate) terminating fragments, (2) sufficiently strong excitation (e.g., through asynchronous release, NMDAr or STF) to overcome sAHP and initiate the next fragment, and (3) sAHP (or another slow-timescale depression) to terminate the NB.

It is important to note that our results do not rule out the possibility that other mechanisms could also generate fragmented NBs. However, we were never able to obtain fragmented NBs in our simulations with the realistic *in silico* model without STD. Nevertheless, other mechanisms not included in our model, such as specific ion-channels or topological influences, could result in fragmented NBs, too. Pradeepan et al. identified increased adaptive currents in RTT neurons and speculated that this could make the neurons more prone to exhibit fragmented NBs (Pradeepan et al., 2024). Some fast afterhyperpolarizing (AHP) or medium AHP (mAHP) currents can depress the firing activity on a relatively short timescale (2–100 ms) (Storm 1989), suggesting candidate mechanisms for fragmented NBs. To test this hypothesis, we included a spike-triggered mAHP current in our computational model instead of STD. Indeed, this mAHP current could also induce fragmented NBs (Figure S4). However, the mAHP current needed to be relatively large (60 times higher conductance compared to sAHP), which is not in line with some literature (Storm 1990). In contrast, the strength of STD we used to obtain fragments was as measured in literature (Markram et al., 1998). Moreover, pharmacological blocking of currents underlying the mAHP resulted in the appearance of fragmented NBs and recapitulation of the GEFS+ and DS phenotypes *in vitro* (Van Hugte et al., 2023). We therefore suspect that STD is a more likely candidate to underlie fragmented NBs than mAHP. Nevertheless, other mechanisms inducing fast-timescale depression may be involved when fragmented NBs are observed *in vitro*.

We revealed candidate mechanisms that underlie the emergence of fragmented NBs. This can help us understand the driving cause of the phenotypes we observe in patient-derived excitatory neuronal networks. In KS networks, fragments could be abolished by applying an NMDAr antagonist, suggesting that enhanced NMDAr function induced the fragmented NBs in those networks (Frega et al., 2019). In DS networks, reduced spontaneous sEPSC

amplitudes and frequencies were observed, which is hypothesized to be caused by homeostatic synaptic downscaling in response to elevated network activity (Doorn et al., 2023). Cohen et al. (Cohen and Segal 2009) showed that in neuronal networks where the activity was artificially elevated, neurons expressed strong STD that was not seen in untreated neurons. This suggests that homeostatic synaptic downscaling could enhance STD, which might cause the fragmented NBs in DS networks, as well as GEFS+ networks, which show a similar phenotype (Van Hugte et al., 2023). In both GEFS+ and DS networks, the number of fragments could be increased by elevating the temperature (Van Hugte et al., 2023). It has been shown in hippocampal slices that increasing the temperature enhances the amount of STF while leaving the amount of STD unaltered (Klyachko and Stevens 2006). Thus, at higher temperatures in GEFS+ and DS networks, strong STD due to homeostatic downscaling may remain while STF increases, promoting the initiation of subsequent fragments as observed in our simulations of NBs with multiple fragments. Moreover, sAHP has been suggested to be lowered in these networks (Doorn et al., 2023), which would allow the occurrence of multiple fragments in combination with enhanced STD (Figure S1).

To conclude, using *in silico* computational models and *in vitro* experiments, we show that enhanced STD is sufficient for the emergence of fragmented NBs. Asynchronous neurotransmitter release acts on STD, causing fragmentation, but it moreover causes left-over excitation after NB termination, allowing the initiation of many more fragments. These multiple fragments could also be induced with sufficiently strong NMDAr currents or STF, in combination with sufficient STD.

EXPERIMENTAL PROCEDURES

In silico modeling

All simulations were performed with the Brian2 simulator (Stimberg et al., 2019) in a Python 3.9 environment. Differential equations were integrated using either the exponential Euler or Euler forward method.

Realistic in silico *model*

The realistic *in silico* model is described in the study by Doorn et al., (2023). In short, it consists of 100 HH-type neurons with voltage-gated sodium and potassium channels, as well as leaky channels and sAHP channels, modeled as a potassium channel whose conductance increases upon action potential firing. The neurons are heterogeneously excitable through a variable external input current, accounting for intrinsic differences. Additionally, the neurons receive noisy fluctuations of their membrane potential to mimic the effect of sEPSCs or other noise components. Neurons are connected to a subset of other neurons through synapses with models of AMPArs, which open immediately upon arrival of a pre-synaptic spike and decay rapidly, and NMDArs, which open and close slowly and are blocked by magnesium ions that are removed upon depolarization of the post-synaptic neuron. The strengths of the synapses vary due to heterogeneous weights (w), drawn from a normal distribution. These weights are further modulated by STD, following the Markram-Tsodysk model (Markram et al., 1998). The model is based on the concept of synaptic resources, of which only a fraction, x, is available, and of which the release probability ($u(t)$) increases upon every pre-synaptic spike. In our original model, we keep $u(t)$ at a value of 1 to only model STD and not STF. The synaptic weight w_j is multiplied by x_j, where x_j obeys:

$$\frac{dx_j}{dt} = \frac{1 - x_j}{\tau_D} - Ux_j\sum_k \delta\left(t - t_j^k - \Delta\right), \quad \text{(Equation 1)}$$

where τ_D is the time constant of STD, and U is the strength of STD.

The parameter values of the model used for all simulations can be found in the Table S1.

Minimal in silico *model*

The minimal model consists of 100 EIF neurons, where the membrane potential is described by:

$$C\frac{dV}{dt} = g_L(E_L - V) + g_L\Delta_T \exp\left(\frac{V - V_T}{\Delta_T}\right) - I_{syn} - I_{sAHP} - I_{noise}, \quad \text{(Equation 2)}$$

with the reset condition that if $V > V_{thres}$ then $V = V_{reset}$. V is the membrane potential, C is the membrane capacitance, E_L is the reversal potential of the leak current, and g_L is the corresponding conductance. I_{sAHP} and I_{noise} are identical to the realistic model, and the synaptic current I_{syn} is given by:

$$I_{syn} = \overline{g}_{syn}(V_m - E_{syn})\sum_{j=1}^{N_E} w_j s_j, \quad \frac{ds_j}{dt} = -\frac{s^j}{\tau_{syn}} + \sum_k \delta\left(t - t_j^k\right), \quad \text{(Equation 3)}$$

where $\overline{g}_{syn}$ is the maximal synaptic conductance when synaptic channels are opened, E_{syn} is the synaptic reversal potential, w_j is the same as in the realistic model, and s is the fraction of open channels that increases upon every pre-synaptic spike at t_j^k and then decays with time constant τ_{syn}.

The neurons in the model are all identical, as well as the synapses. These synapses also undergo STD as described earlier. All parameter values used for simulations with the minimal model can be found in the provided Python code.

STF and asynchronous release

To model STF in the model, we use the release probability $u(t)$ as described by Markram and Tsodyks (Markram et al., 1998), which, together with the aforementioned model for STD, forms the short-term plasticity (STP) model:

$$\frac{dx_j}{dt} = \frac{1 - x_j}{\tau_D} - u_j x_j \sum_k \delta\left(t - t_j^k - \Delta\right),$$

$$\frac{du_j}{dt} = \frac{U - u_j}{\tau_F} + U(1 - u_j)\sum_k \delta\left(t - t_j^k - \Delta\right), \quad \text{(Equation 4)}$$

where τ_F is the recovery time constant of facilitation and τ_D is the time constant of depression. The synaptic weight is multiplied by

both u_j and x_j. Thus, at every pre-synaptic spike, u_j increases with $U(1 - u)$, and an amount of $u_j x_j$ neurotransmitters is released and subtracted from x_j.

To model asynchronous release, we used an extension of the STP model as described in the study by Wang et al. (Wang et al., 2015). In this extension, u_{sr} describes the release probability of synchronous release as described in Equation 4, and u_{ar} is the probability rate of asynchronous release. Research has shown that synchronous and asynchronous release are mediated by different Ca^{2+} sensors with distinct association and dissociation rates with Ca^{2+} (Wen et al., 2010). Therefore, both releases have different recovery time constants (dissociation rates), τ_{sr} and τ_{ar}, and different saturation levels U_{sr} and U_{ar}. Based on the literature, we also assume that synchronous and asynchronous releases are competing for the same vesicle pool (Wen et al., 2010). We thus use one variable x as described in equation 4. While synchronous release is time-locked to the arrival of a pre-synaptic spike, asynchronous release is very stochastic. We therefore model it using a binomial process. We call x_0 the amount of neurotransmitter in one vesicle, making $x(t)/x_0$ the maximum number of releasable vesicles. In a time interval $[t, t+dt]$, the release probability of a single vesicle is given by $u_{ar}dt$. We assume the amount of asynchronous release events $n(t)$ follows a binomial distribution $\mathcal{B}(\lfloor x(t)\ /x_0 \rfloor, u_{\mathrm{ar}}(t)dt)$. The overall asynchronous release rate is then:

$$q_{\mathrm{ar}}(t)\mathrm{d}t \ = \ x_0 n_{\mathrm{ar}}(t). \qquad \text{(Equation 5)}$$

Because we cannot model a binomial distribution in Brian2, we approximate it with a normal distribution cutoff at the borders of the binomial distribution. The parameters used for all simulations can be found in Table S1.

MEA recordings and data analysis

We used MEA recordings performed on neuronal networks derived from hiPSCs of control and patients with GEFS+ and DS (FAM001 GEFS and FAM001 DRAV described in the study by Van Hugte et al., 2023) and control and a patient with KS (C_{MOS} and KS_{MOS} described in the study by Frega et al., 2019) (data in Figures 1B and 1C). In addition, we used a representative MEA recording performed on neuronal networks derived from hiPSCs of control and patients with RTT (described in the study by Pradeepan et al., 2024) (data in Figures 4A and 4B). To evaluate the effect of enhanced STD *in vitro*, we performed experiments in which we differentiated neuronal networks from hiPSCs of a healthy individual (Mossink et al., 2021) (data in Figure 3). We performed recordings using the Multiwell-MEA system on day *in vitro* (DIV) 35 (Multichannel Systems, MCS GmbH, Reutlingen, Germany). MEA devices are composed of 24 independent wells with embedded micro-electrodes (i.e., 12 electrodes/well, 80 μm in diameter and spaced 300 μm apart). During recording, the temperature was maintained at 37°C, and a slow flow of humidified gas (5% CO_2 and 95% ambient air) was applied onto the MEA plate. Every electrode recorded voltages with a sampling frequency of 10 kHz. Also from the *in silico* model, "virtual electrode" recordings were sampled at 10 kHz and then handled identically to experimental data. Wells that did not show electrical activity, that did not show NBs, or that showed unevenly distributed neurons under the microscope were excluded (Mossink et al., 2021).

Signals were filtered between 100 and 3,500 Hz using a fifth-order Butterworth filter. We detected spikes using an amplitude threshold-based method, where the threshold was four times the root mean square of the electrode signal. The network firing rate was computed by binning spikes at all electrodes in 25 ms time bins. We then smoothed this network firing rate by convolution with a Gaussian kernel. To detect NBs, we employed two thresholds on this smoothed firing rate to start and stop the NB, set to 1/4th and 1/100th of the maximum firing rate, respectively. Additionally, 30% of the active electrodes (i.e., electrodes with an average firing rate above 0.02 Hz) had to be firing during the NB, and the firing rate should remain above the NB start- or stop-threshold for 50 ms to start or end NB detection. Fragments were detected using a peak detection algorithm on the smoothed network firing rate where peaks should have a minimal height of 1/16th of the maximum firing rate and a minimal prominence of 1/10th of the maximum firing rate. The number of fragments per NB was then defined as the number of detected peaks within the duration of the NB.

We defined four features that were representative of the spontaneous network activity. Specifically, the average number of fragments per NB (#Fragments/NB) was calculated by summing all detected fragments and dividing them by the number of detected NBs. Additionally, the NBR (the average number of NBs per minute), NBD (the mean duration of NBs during recording), and CV_{IBI} (the coefficient of variation of the inter-burst intervals) were calculated.

Dynasore experiments and modeling

We used a previously characterized *Ngn2*-positive hiPSC line that was infected, according to a previously published protocol (Frega et al., 2017), with lentiviral constructs encoding *rtTA* combined with *Ngn2* to generate doxycycline-inducible excitatory neurons (Mossink et al., 2021; Frega et al., 2019). The hiPSC line was generated from fibroblasts of a healthy individual (male, 30 years old) via episomal reprogramming (Mandegar et al., 2016). We received the *Ngn2*-positive hiPSC line at passage 10 in frozen vials, kindly provided by Prof. Nadif Kasri (Radboud University Medical Center, The Netherlands). The research was conducted in accordance with the principles embodied in the Declaration of Helsinki and local statutory requirements. The genetically modified organism approval under which the line (name: IPS16-00016) has been used is IG22-071. Karyotypes of hiPSC line were verified, and hiPSC line was tested for pluripotency and genomic integrity based on single-nucleotide polymorphism arrays (Mossink et al., 2021; Frega et al., 2019). Cells were thawed in E8 flex medium (Gibco, #A2858501) supplemented with RevitaCel (Thermo Fisher Scientific, #A2644501), puromycin (0.5 μg/mL, Sigma-Aldrich, #P9620), and G418 (50 μg/mL, Sigma-Aldrich, #G8168) on 6-well plates pre-coated with Geltrex (Thermo Fisher Scientific #A1413302) and were maintained in this medium at 37°C/5% CO_2. The medium was refreshed every 2–3 days, and cells were passaged twice per week using an enzyme-free reagent (ReLeSR, STEMCELL Technologies, #05872) and not kept for more than 10 passages. Every month, cells were checked for mycoplasma contamination using MycoAlert PLUS (Lonza, #LT07-703). Cells were frozen at 80% confluency within E8 flex medium with 20% DMSO supplemented

with RevitaCel, puromycin (0.5 μg/mL), and G418 (50 μg/mL). We differentiated hiPSCs into excitatory cortical layer 2/3 neurons on MEA through doxycycline-inducible overexpression of *Neurogenin 2* (*Ngn2*) as described previously (Frega et al., 2017; Mossink et al., 2021) (Figure 1A). In short, *Ngn2*-positive hiPSCs were cultured on Geltrex (Thermo Fischer Scientific, #A1413302) in E8 flex medium, supplemented with puromycin (0.5 μg/mL) and G418 (50 μg/mL) at 37°C/5% CO_2. On DIV 0, *Ngn2*-positive hiPSCs were co-plated as single cells in 24-well MEAs, pre-coated with poly-*l*-ornithine (50 μg/mL, Sigma-Aldrich, #P4957) and human laminin (5 μg/mL, BioLamina, #LN521). To promote neuronal maturation, astrocytes obtained from cortices of newborn (P1) Wistar rats were added to hiPSC cultures in a 1:1 ratio on DIV 2. All surgical and experimental procedures regarding animal primary cell lines followed Dutch and European laws and guidelines and were approved by the Centrale Commissie Dierproeven (AVD11000202115663). On DIV 3, the medium was changed to Neurobasal medium (Gibco, #21103049) supplemented with DOX (4 μg/mL, Sigma-Aldrich, #D9891), B-27 (Thermo Fisher Scientific, #17504044), glutaMAX (2 mM, Thermo Fisher Scientific, #35050061), primocin (0.1 μg/mL, InvivoGen, #ant-pm-05), neurotrophin-3 (10 ng/mL, STEMCELL, #78074), and brain-derived neurotrophic factor (10 ng/mL, STEMCELL, #78005). Cytosine β-D-arabinofuranoside (2 μM, Sigma-Aldrich, #C1768) was added to eliminate proliferating cells. Starting from this day, half of the medium was changed three times a week. From DIV 10 onward, 2.5% fetal bovine serum (Sigma-Aldrich, #F9665) was added to support astrocyte viability. DOX was removed after DIV 14. Cells were maintained in an incubator at 37°C with 80% humidity and 5% CO_2 until the experiment on DIV 35.

Culturing and experiments were performed with two independent neuronal preparations. Baseline spontaneous activity was recorded for 5 min. Immediately following baseline recording, a pharmacological agent (10 μM Dynasore [Sigma-Aldrich, #D7693, diluted in DMSO] or 0.1% DMSO) was added to each well, and 20 min of post-treatment activity was recorded. Only the last 5 min of recording post-treatment were used for analysis.

In the computational model, the effect of Dynasore was modeled by simulating a network for 5 min, then increasing U from 0.006 to 0.035, while keeping all other parameters identical, and then continuing the simulation for 5 min. To explore the effect of increasing U across all possible model parameter configurations, we performed 120,000 simulations of 3 min with different values of the nine most critical model parameters (σ, g_{Na}, g_K, g_{sAHP}, S, δ, Connection prob, τ_D, and U). Next, we identified simulations that exhibited all key features (#Fragments, NBR, NBD, and CV_{IBI}) within the ranges observed in the basal *in vitro* neuronal networks (Figure 3E, e.g., NBR between 1.8 and 4.8 NBs/min). Subsequently, in these simulations, U was increased by 50% and the effect was observed for another 3 min, after which features were again quantified.

Statistics

We performed statistical analysis using GraphPad Prism 5 (GraphPad Software, Inc., CA, USA). We checked for normal distributions using a Kolmogorov-Smirnov test. We compared the number of fragments in NBs of patient-derived neuronal networks using a Kruskal-Wallis test with Dunn's multiple comparisons test, because not all data were normally distributed. To compare the effect of Dynasore to vehicle, and to its *in silico* counterpart, we used a two-way ANOVA with uncorrected Fisher's LSD for multiple comparisons. p values <0.05 were considered significant in all cases. All data points, statistics, and p values can be found in Table S2.

RESOURCE AVAILABILITY

Lead contact

Further information and requests for resources and reagents should be directed to and will be fulfilled by the lead contact, Nina Doorn (n.doorn-1@utwente.nl).

Materials availability

All reagents or cell lines used in this study are available from the lead contact upon request with a completed Materials Transfer Agreement.

Data and code availability

The Python code to run simulations *in silico* and analyze MEA recordings as well as all newly generated data is published on GitLab (https://gitlab.utwente.nl/m7706783/fb_model). All individual values shown in figures and the corresponding p values can be found in Table S2.

We followed FAIR and CARE data management principles in our study.

ACKNOWLEDGMENTS

This work was supported by the ZonMw (Netherlands Organisation for Health Research and Development) BRAINmodel PSIDER program 10250022110003 (to M.F.). We thank Eline van Hugte, Nael Nadif Kasri, and James Ellis for providing MEA recordings from patient-derived *in vitro* neuronal networks.

AUTHOR CONTRIBUTIONS

N.D. developed the *in silico* models and designed and performed *in silico* experiments. N.D., E.J.H.F.V., and M.R.L. designed, prepared, and performed *in vitro* experiments. N.D. designed and performed data analyses. N.D. wrote the manuscript with input from all authors. M.F. and M.J.A.M.v.P. provided conceptualization and intellectual content. M.F. conceived and supervised the project.

DECLARATION OF INTERESTS

The authors declare no competing interests.

SUPPLEMENTAL INFORMATION

Supplemental information can be found online at https://doi.org/10.1016/j.stemcr.2024.09.001.

Received: March 1, 2024
Revised: August 30, 2024
Accepted: September 2, 2024
Published: October 3, 2024

REFERENCES

Cohen, D., and Segal, M. (2009). Homeostatic presynaptic suppression of neuronal network bursts. J. Neurophysiol. *101*, 2077–2088. https://doi.org/10.1152/JN.91085.2008.

Compte, A., Brunel, N., Goldman-Rakic, P.S., and Wang, X.J. (2000). Synaptic Mechanisms and Network Dynamics Underlying Spatial Working Memory in a Cortical Network Model. Cereb. Cortex *10*, 910–923. https://doi.org/10.1093/CERCOR/10.9.910.

Dao Duc, K., Lee, C.Y., Parutto, P., Cohen, D., Segal, M., Rouach, N., and Holcman, D. (2015). Bursting Reverberation as a Multiscale Neuronal Network Process Driven by Synaptic Depression-Facilitation. PLoS One *10*, e0124694. https://doi.org/10.1371/JOURNAL.PONE.0124694.

Doorn, N., van Hugte, E.J.H., Ciptasari, U., Mordelt, A., Meijer, H.G.E., Schubert, D., Frega, M., Nadif Kasri, N., and van Putten, M.J.A.M. (2023). An in silico and *in vitro* human neuronal network model reveals cellular mechanisms beyond NaV1.1 underlying Dravet syndrome. Stem Cell Rep. *18*, 1686–1700. https://doi.org/10.1016/J.STEMCR.2023.06.003.

Douthitt, H.L., Luo, F., McCann, S.D., and Meriney, S.D. (2011). Dynasore, an inhibitor of dynamin, increases the probability of transmitter release. Neuroscience *172*, 187–195. https://doi.org/10.1016/J.NEUROSCIENCE.2010.10.002.

Fardet, T., Ballandras, M., Bottani, S., Métens, S., and Monceau, P. (2018). Understanding the generation of network bursts by adaptive oscillatory neurons. Front. Neurosci. *12*, 41. https://doi.org/10.3389/FNINS.2018.00041.

Frega, M., Linda, K., Keller, J.M., Gümüş-Akay, G., Mossink, B., van Rhijn, J.R., Negwer, M., Klein Gunnewiek, T., Foreman, K., Kompier, N., et al. (2019). Neuronal network dysfunction in a model for Kleefstra syndrome mediated by enhanced NMDAR signaling. Nat. Commun. *10*, 4928. https://doi.org/10.1038/s41467-019-12947-3.

Frega, M., van Gestel, Linda, K., van der Raadt, J., Keller, J., Van Rhijn, J., Schubert, D., Albers, C.A., and Nadif Kasri, N. (2017). Rapid Neuronal Differentiation of Induced Pluripotent Stem Cells for Measuring Network Activity on Micro-electrode Arrays. J. Vis. Exp. *119*, e54900. https://doi.org/10.3791/54900.

Gabriele, M., Vitriolo, A., Cuvertino, S., Pereira, M.F., Franconi, C., Germain, P.L., Capocefalo, D., Castaldi, D., Tenderini, E., Bèchet, N.B., et al. (2021). KMT2D haploinsufficiency in Kabuki syndrome disrupts neuronal function through transcriptional and chromatin rewiring independent of H3K4-monomethylation. Preprint at bioRxiv. https://doi.org/10.1101/2021.04.22.440945.

Hedegaard, M., Hansen, K.B., Andersen, K.T., Bräuner-Osborne, H., and Traynelis, S.F. (2012). Molecular pharmacology of human NMDA receptors. Neurochem. Int. *61*, 601–609. https://doi.org/10.1016/J.NEUINT.2011.11.016.

Hofmann, M.E., and Andresen, M.C. (2017). Dynasore blocks evoked release while augmenting spontaneous synaptic transmission from primary visceral afferents. PLoS One *12*, e0174915. https://doi.org/10.1371/JOURNAL.PONE.0174915.

Hua, Y., Woehler, A., Kahms, M., Haucke, V., Neher, E., and Klingauf, J. (2013). Blocking Endocytosis Enhances Short-Term Synaptic Depression under Conditions of Normal Availability of Vesicles. Neuron *80*, 343–349. https://doi.org/10.1016/J.NEURON.2013.08.010.

Huang, C.H., Huang, Y.T., Chen, C.C., and Chan, C.K. (2017). Propagation and synchronization of reverberatory bursts in developing cultured networks. J. Comput. Neurosci. *42*, 177–185. https://doi.org/10.1007/S10827-016-0634-4.

Jackman, S.L., and Regehr, W.G. (2017). The Mechanisms and Functions of Synaptic Facilitation. Neuron *94*, 447–464. https://doi.org/10.1016/J.NEURON.2017.02.047.

Kaeser, P.S., and Regehr, W.G. (2014). Molecular Mechanisms for Synchronous, Asynchronous, and Spontaneous Neurotransmitter Release. Annu. Rev. Physiol. *76*, 333–363. https://doi.org/10.1146/ANNUREV-PHYSIOL-021113-170338.

Klein Gunnewiek, T., Van Hugte, E.J.H., Frega, M., Guardia, G.S., Foreman, K.B., Panneman, D., Mossink, B., Linda, K., Keller, J.M., Schubert, D., et al. (2020). m.3243A > G-Induced Mitochondrial Dysfunction Impairs Human Neuronal Development and Reduces Neuronal Network Activity and Synchronicity. Cell Rep. *31*, 107538. https://doi.org/10.1016/j.celrep.2020.107538.

Klein Gunnewiek, T.M., Verboven, A.H.A., Pelgrim, I., Hogeweg, M., Schoenmaker, C., Renkema, H., Beyrath, J., Smeitink, J., de Vries, B.B.A., 't Hoen, P.B.A.C., et al. (2021). Sonlicromanol improves neuronal network dysfunction and transcriptome changes linked to m.3243A>G heteroplasmy in iPSC-derived neurons. Stem Cell Rep. *16*, 2197–2212. https://doi.org/10.1016/J.STEMCR.2021.07.002.

Klyachko, V.A., and Stevens, C.F. (2006). Temperature-Dependent Shift of Balance among the Components of Short-Term Plasticity in Hippocampal Synapses. J. Neurosci. *26*, 6945–6957. https://doi.org/10.1523/JNEUROSCI.1382-06.2006.

Lau, P.M., and Bi, G.Q. (2005). Synaptic mechanisms of persistent reverberatory activity in neuronal networks. Proc. Natl. Acad. Sci. USA *102*, 10333–10338. https://doi.org/10.1073/PNAS.0500717102.

Linda, K., Lewerissa, E.I., Verboven, A.H.A., Gabriele, M., Frega, M., Klein Gunnewiek, T.M., Devilee, L., Ulferts, E., Hommersom, M., Oudakker, A., et al. (2022). Imbalanced autophagy causes synaptic deficits in a human model for neurodevelopmental disorders. Autophagy *18*, 423–442. https://doi.org/10.1080/15548627.2021.1936777.

Mandegar, M.A., Huebsch, N., Frolov, E.B., Shin, E., Truong, A., Olvera, M.P., Chan, A.H., Miyaoka, Y., Holmes, K., Spencer, C.I., et al. (2016). CRISPR Interference Efficiently Induces Specific and Reversible Gene Silencing in Human iPSCs. Cell Stem Cell *18*, 541–553. https://doi.org/10.1016/J.STEM.2016.01.022.

Marchetto, M.C., Belinson, H., Tian, Y., Freitas, B.C., Fu, C., Vadodaria, K., Beltrao-Braga, P., Trujillo, C.A., Mendes, A.P.D., Padmanabhan, K., et al. (2017). Altered proliferation and networks in neural cells derived from idiopathic autistic individuals. Mol. Psychiatr. *22*, 820–835. https://doi.org/10.1038/MP.2016.95.

Markram, H., Wang, Y., and Tsodyks, M. (1998). Differential signaling via the same axon of neocortical pyramidal neurons. Proc. Natl. Acad. Sci. USA *95*, 5323–5328. https://doi.org/10.1073/pnas.95.9.5323.

Masquelier, T., and Deco, G. (2013). Network bursting dynamics in excitatory cortical neuron cultures results from the combination of

different adaptive mechanisms. PLoS One *8*, e75824. https://doi.org/10.1371/JOURNAL.PONE.0075824.

Mossink, B., Verboven, A.H.A., van Hugte, E.J.H., Klein Gunnewiek, T.M., Parodi, G., Linda, K., Schoenmaker, C., Kleefstra, T., Kozicz, T., van Bokhoven, H., et al. (2021). Human neuronal networks on micro-electrode arrays are a highly robust tool to study disease-specific genotype-phenotype correlations *in vitro*. Stem Cell Rep. *16*, 2182–2196. https://doi.org/10.1016/J.STEMCR.2021.07.001.

Newton, A.J., Kirchhausen, T., and Murthy, V.N. (2006). Inhibition of dynamin completely blocks compensatory synaptic vesicle endocytosis. Proc. Natl. Acad. Sci. USA *103*, 17955–17960. https://doi.org/10.1073/pnas.0606212103.

Pradeepan, K.S., McCready, F.P., Wei, W., Khaki, M., Zhang, W., Salter, M.W., Ellis, J., and Martinez-Trujillo, J. (2024). Calcium dependent hyperexcitability in human stem cell derived Rett syndrome neuronal networks. Biol. Psychiatr. Global Open Sci. *4*, 100290. https://doi.org/10.1016/J.BPSGOS.2024.100290.

Stimberg, M., Brette, R., and Goodman, D.F. (2019). Brian 2, an intuitive and efficient neural simulator. Elife *8*, e47314. https://doi.org/10.7554/eLife.47314.

Storm, J.F. (1989). An after-hyperpolarization of medium duration in rat hippocampal pyramidal cells. J. Physiol. *409*, 171–190. https://doi.org/10.1113/JPHYSIOL.1989.SP017491.

Storm, J.F. (1990). Chapter 12 Potassium currents in hippocampal pyramidal cells. Prog. Brain Res. *83*, 161–187. https://doi.org/10.1016/S0079-6123(08)61248-0.

Van Hugte, E.J.H., Lewerissa, E.I., Wu, K.M., Scheefhals, N., Parodi, G., van Voorst, T.W., Puvogel, S., Kogo, N., Keller, J.M., Frega, M., et al. (2023). SCN1A-deficient excitatory neuronal networks display mutation- specific phenotypes. Brain *146*, 5153–5167. https://doi.org/10.1093/BRAIN/AWAD245.

Volman, V., Gerkin, R.C., Lau, P.M., Ben-Jacob, E., and Bi, G.Q. (2007). Calcium and synaptic dynamics underlying reverberatory activity in neuronal networks. Phys. Biol. *4*, 91–103. https://doi.org/10.1088/1478-3975/4/2/003.

Wagenaar, D.A., Pine, J., and Potter, S.M. (2006). An extremely rich repertoire of bursting patterns during the development of cortical cultures. BMC Neurosci. *7*, 11–18. https://doi.org/10.1186/1471-2202-7-11.

Wang, S., Rhijn, J.R.v., Akkouh, I., Kogo, N., Maas, N., Bleeck, A., Ortiz, I.S., Lewerissa, E., Wu, K.M., Schoenmaker, C., et al. (2022). Loss-of-function variants in the schizophrenia risk gene SETD1A alter neuronal network activity in human neurons through the cAMP/PKA pathway. Cell Rep. *39*, 110790. https://doi.org/10.1016/J.CELREP.2022.110790.

Wang, T., Yin, L., Zou, X., Shu, Y., Rasch, M.J., and Wu, S. (2015). A phenomenological synapse model for asynchronous neurotransmitter release. Front. Comput. Neurosci. *9*, 153. https://doi.org/10.3389/FNCOM.2015.00153.

Wang, X.J. (1999). Synaptic Basis of Cortical Persistent Activity: the Importance of NMDA Receptors to Working Memory. J. Neurosci. *19*, 9587–9603. https://doi.org/10.1523/JNEUROSCI.19-21-09587.1999.

Wen, H., Linhoff, M.W., McGinley, M.J., Li, G.L., Corson, G.M., Mandel, G., and Brehm, P. (2010). Distinct roles for two synaptotagmin isoforms in synchronous and asynchronous transmitter release at zebrafish neuromuscular junction. Proc. Natl. Acad. Sci. USA *107*, 13906–13911. https://doi.org/10.1073/PNAS.1008598107.

Stem Cell Reports, Volume *19*

Supplemental Information

Breaking the burst: Unveiling mechanisms behind fragmented network bursts in patient-derived neurons

Nina Doorn, Eva J.H.F. Voogd, Marloes R. Levers, Michel J.A.M. van Putten, and Monica Frega

Supplemental Figures

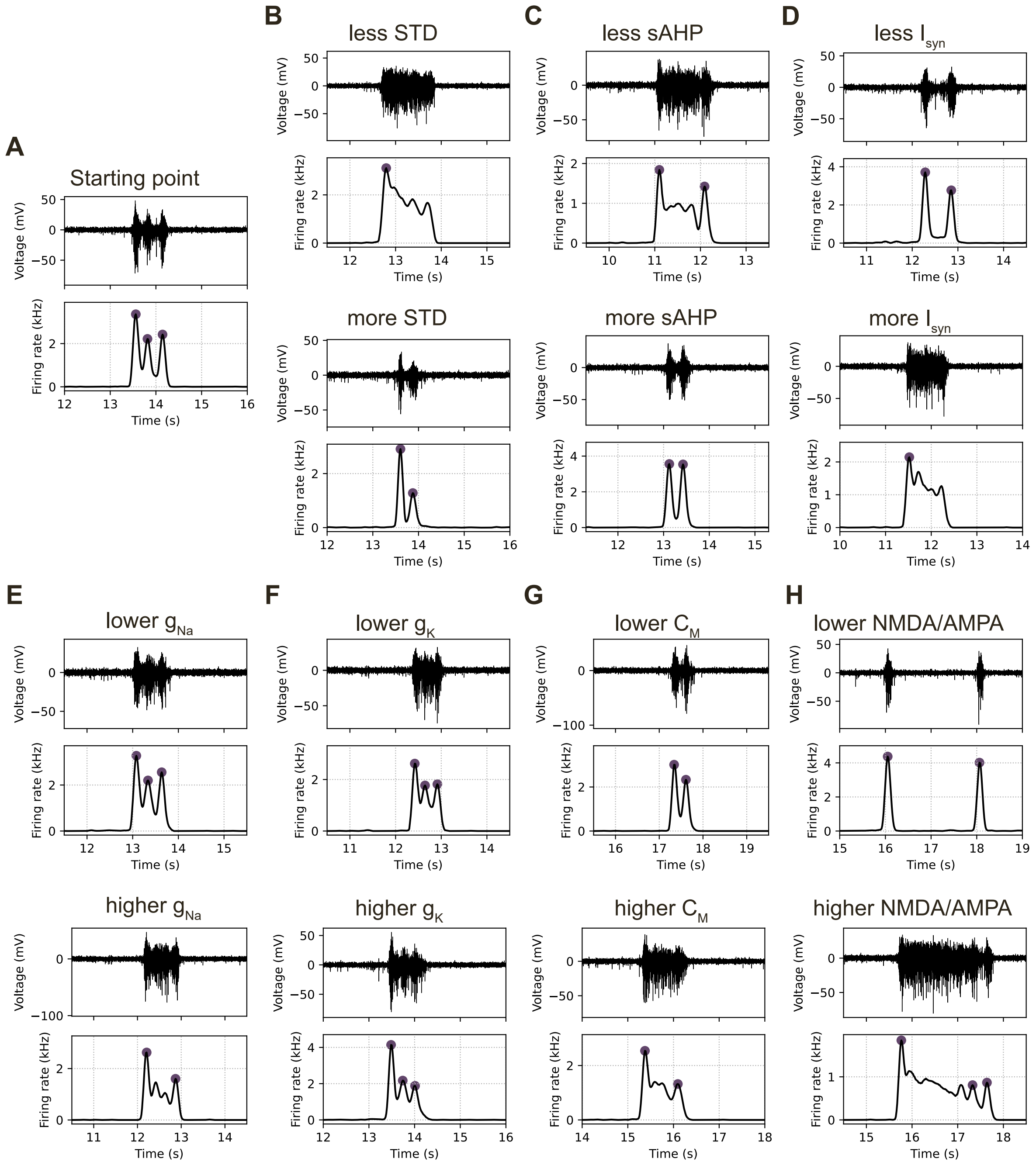

Figure S1: **The effect of model parameters on the number and shape of fragments in simulations.**
A) Starting point of parameter configuration resulting in simulations with network bursts (NBs) consisting of three fragments. **B)** The effect of less or more STD on the simulated NBs. Modeled as an increase or decrease in parameter U. **C)** The effect of less or more sAHP current on the simulated NBs. Modeled as an increase or decrease in the conductance of the sAHP channels. **D)** The effect of less or more synaptic current (I_{syn}) on the simulated NBs. Modeled as an increase or decrease in the conductance of all synaptic channels. **E)** The effect of a higher or lower maximum voltage-gated sodium channel conductance on the simulated NBs. **F)** The effect of a higher or lower maximum voltage-gated potassium channel conductance on the simulated NBs. **G)** The effect of a higher or lower neuronal membrane capacitance on the simulated NBs. **H)** The effect of a higher or lower NMDA/AMPA ratio on the simulated NBs. Modeled as a shift in the relative maximum conductances of the NMDA and AMPA channels (corresponding to the relative amount of channels or their synaptic strengths).

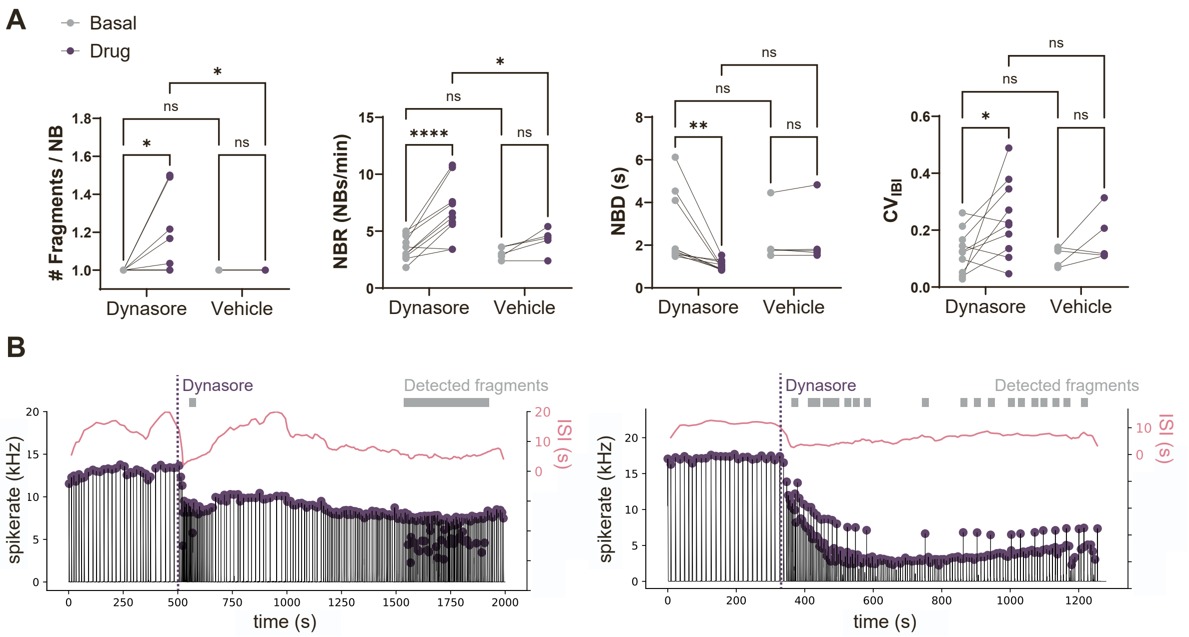

Figure S2: **Dynasore affects neuronal network activity differently from vehicle and the effect varies throughout measurement.**

A) The effect on the neuronal network activity when either Dynasore (10 μM) (n=10) or vehicle (0.1% DMSO) (n=5) is applied to the network. Figure shows the change in the average number of fragments per network burst (NB) (# Fragments / NB), the NB rate (NBR), the NB duration (NBD), and the coefficient of variation of the inter burst intervals (CV_{IBI}). ns $P>0.05$, * $P<0.05$, ** $P<0.01$, **** $P<0.0001$, 2way ANOVA with uncorrected Fisher's LSD for multiple comparisons was performed between groups. **B)** Two examples of recordings of healthy neuronal network activity in which Dynasore was added (dashed line). Shown is the network firing rate in black with detected peaks in purple. In both examples, a decrease in the network firing rate can be seen upon Dynasore application, together with a decrease in the inter-spike interval (ISI, pink line) and some fragment detections (grey bar). Then the ISI slowly increases again while the amount of fragment detections drops to zero. Towards the end of the recording, the amount of fragment detections again increases, illustrating the variable effect Dynasore has on the *in vitro* neuronal network over time.

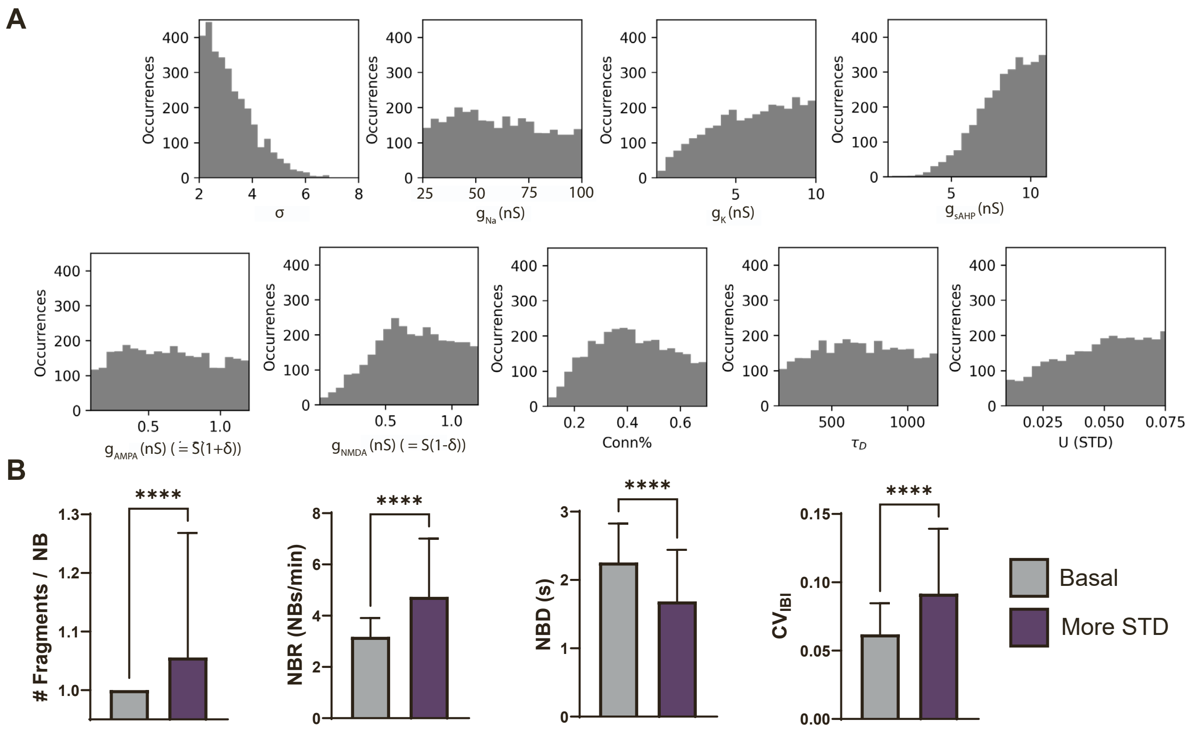

Figure S3: **The effect of increasing STD *in silico* with different starting parameter configurations.**

A) Histograms of the parameter values resulting in simulations consistent with experimental data. **B)** Grey shows data features of 3114 basal simulations with different model parameter configurations. Purple shows data features when the strength of STD (U) was increased by 50% in these simulations. Data shows mean $\pm$ sd. Wilcoxon matched-pairs signed rank test was performed between groups. **** P$<$0.0001.

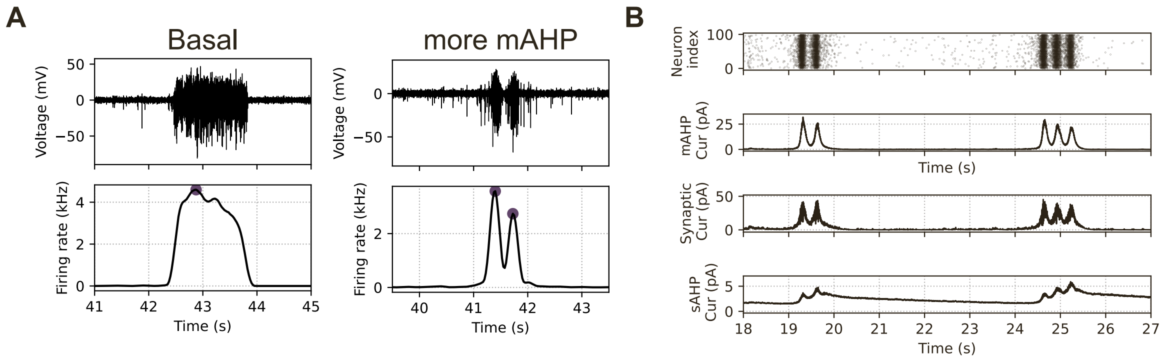

Figure S4: **Fragmented NBs can also arise from the interplay between mAHP and sAHP.** **A)** Top: Voltage recorded at a single virtual electrode. Bottom: Network firing rate with detected fragments (purple dots). Left: Basal situation with no STD and no mAHP. Right: Simulation of the same network but with increased mAHP. **B)** Mechanism causing fragmented NBs: Recurrent excitation (third panel) starts an NB causing an increase in firing (top panel). This increase in firing causes an increase in both the mAHP (second panel) and sAHP (fourth panel) currents. This lowers the firing rate, terminating the fragment. Then, mAHP recovers quickly, allowing left-over excitation to initiate the next fragment until sAHP (recovering slowly) is high enough to terminate the entire NB. mAHP is modeled identically to sAHP (conductance increases at every spike and decays exponentially), but with a time constant of 50 ms instead of 8000 ms and a conductance of 350 nS instead of 6 nS.

Supplemental Tables

Table S1: Values of the parameters of the realistic *in silico* model used for the results and examples shown in the Figures.

Parameter	Unit	Fig 2B	Fig 3C,D	Fig 4C,D	Fig 4G,H	Fig 4I,J
Connection prob		0.2	0.2	0.2	0.3	0.2
Membrane area	μm^{-2}	300	300	300	300	300
C	μF $\cdot$ cm^{-2}	2	2	2	1	2
E_{L}	mV	-39.2	-39.2	-39.2	-39.2	-39.2
E_{K}	mV	-80	-80	-80	-80	-80
E_{Na}	mV	70	70	70	70	70
g_{Na}	mS $\cdot$ cm^{-2}	80	80	80	50	80
g_{K}	mS $\cdot$ cm^{-2}	6.5	6.5	6.5	5	6.5
g_{L}	mS $\cdot$ cm^{-2}	0.3	0.3	0.3	0.3	0.3
Firing threshold	mV	-30.4	-30.4	-30.4	-30.4	-30.4
σ	mV	8	6	5.5	3.5	5.5
g_{sAHP}	nS	5	4	8	5	5
τ_{AHP}	ms	8000	8000	8000	8000	8000
α_{Ca}		0.00035	0.00035	0.00035	0.00035	0.00035
S		0.4	0.25	0.4	1.5	13
δ		0.6	0.6	0.6	0.5	0.6
E_{AMPA}	mV	0	0	0	0	0
E_{NMDA}	mV	0	0	0	0	0
τ_{AMPA}	ms	2	2	2	2	2
$\tau_{\text{NMDA,rise}}$	ms	2	2	2	2	2
$\tau_{\text{NMDA,decay}}$	Ms	100	100	100	100	100
α	kHz	0.5	0.5	0.5	0.5	0.5
τ_{D}	ms	200	250	200	800	1000
U		0.2	6e-3/35e-3	0.01	0.2	0.005
Apply STF	t/f	FALSE	FALSE	FALSE	FALSE	TRUE
τ_{F}	ms	-	-	-	-	1000
Apply Asyn	t/f	FALSE	FALSE	TRUE	FALSE	FALSE
τ_{ar}	ms	-	-	700	-	-
U_{ar}		-	-	0.5	-	-
U_{max}	1/ms	-	-	5e-4/45e-4	-	-
X_0		-	-	5	-	-
